# Effects of different potassium application rates on potato yield and the expression of CIPK8, a candidate gene involved in potassium uptake

**DOI:** 10.3389/fpls.2026.1912167

**Published:** 2026-09-30

**Authors:** Yaoyao Gai, Dong Wang, Yikun Li, Zhiping Li, Shujuan Wang, Wenrui Zhao, Xinyao Shi, Ziyi Zhang

**Affiliations:** 1 College of Life Sciences, Inner Mongolia Agriculture University, Hohhot, Inner Mongolia, China; 2 Inner Mongolia Autonomous Region Agriculture and Animal Husbandry Technology Extension Center, Hohhot, Inner Mongolia, China; 3 Inner Mongolia Xinyu Seed Industry Co., Ltd., Ulanqab, Inner Mongolia, China

**Keywords:** CIPK8, potassium application rate, potassium uptake, potato, subcellular localization, yield

## Abstract

**Introduction:**

Potassium is critical for potato yield, but low use efficiency dueto improper fertilization limits production.

**Methods:**

Here, we investigated the effects of different potassium applicationrates (0, 120, 180, 240, and 300 kg·hm^2^ K?O in 2024; and 0, 180, 240, and300 kg·hm^2^ K_2_O in 2025) on potato yield and CIPK8 expression over twofield seasons.

**Results:**

Yield peaked at 240 kg·hm^2^ (K240), increasing by 23–29% over thecontrol. CIPK8 gene and protein expression followed a unimodal pattern,peaking at 90 days after planting (DAP; tuber bulking stage), with K240inducing >50% higher expression than K0 and significantly higher expressionthan K300. Fluorescence in situ hybridization detected CIPK8 sequences inthe nucleus, while immunofluorescence detected CIPK8 protein in thenucleus, cytoplasm, and membrane.

**Discussion:**

Our results suggest that K240 is associated with the highestCIPK8 expression and potato yield, suggesting CIPK8 as a potential candidateinvolved in potassium uptake regulation. However, further functionalvalidation is needed to establish a causal relationship. This study provides amolecular basis for efficient potassium management in potato.

## 1 Introduction

Potato (*Solanum tuberosum* L.) is one of the world’s most important food crops, serving both as a staple food and a vegetable. With high nutritional value, high yield potential, and broad adaptability, it is a strategic crop for strengthening national food security (Kloosterman et al., 2013; Siamalube et al., 2025). The northern foothills of the Yinshan Mountains in Inner Mongolia represent a typical agro-pastoral ecotone, where the large diurnal temperature range and cool climate are highly suitable for potato cultivation (Yao et al., 2024; Southern et al., 2025). However, the sustainable development of the potato industry faces many challenges, among which soil nutrient management, particularly potassium management, has become a key bottleneck that restricts further improvements in yield and quality (Fan et al., 2025). Recent studies have systematically examined potassium fertilization effects on potato yield and quality across different regions. A meta-analysis by (Zhang et al., 2025) quantified the yield response of potato to potassium application across China, identifying an optimal range that varies with soil type and climate. (Fan et al., 2025) established critical nitrogen and potassium dilution curves for potato in Northeast China, providing a diagnostic tool for nutrient management. In addition, potassium fertilization has been shown to affect storage root initiation, lignin biosynthesis, and tuber quality parameter. These agronomic studies have established the importance of potassium for potato production but have not addressed the underlying molecular mechanisms.

Potassium is an essential macronutrient for plant growth and development, playing critical roles in crop yield formation and stress tolerance enhancement. In potato, potassium is taken up in larger amounts than any other mineral nutrient, directly influencing tuber yield and overall plant vigor (Mostofa et al., 2022; Rawat et al., 2022). Potato is a potassium-loving crop, absorbing more potassium than any other nutrient during its growth cycle, which directly affects tuber yield and is a key factor in determining potassium use efficiency (Deng et al., 2020). In current potato production, improper potassium fertilizer management—including deficiency, excess, and low use efficiency—has led to poor crop growth, yield reduction, and environmental pollution (Ravensbergen et al., 2024; Wei et al., 2024; Southern et al., 2025). An appropriate potassium supply can modulate lignin biosynthesis, promote storage root initiation, and ultimately enhance tuber yield and quality (Lindqvist-Kreuze et al., 2023; Liu et al., 2023). Notably, potatoes are sensitive to chloride ions, so potassium sulfate (K_2_SO_4_) is generally recommended over potassium chloride (KCl) in production (Wilmer et al., 2022).

The CBL-CIPK signaling module (**Figure 1**) plays a central role in plant potassium nutrition. The CIPK (CBL-interacting protein kinase) family belongs to the serine/threonine protein kinase superfamily, and its interacting CBL proteins serve as calcium sensors that perceive Ca^2+^ signals and form the CIPK-CBL complex (Sanyal et al., 2016). This module finely regulates K^+^ uptake, transport, and compartmentalization in response to changes in intracellular calcium and is widely involved in abiotic stress responses (salt, cold, drought, pH) and ABA signaling (Pandey and Mahiwal, 2020; You et al., 2023). For example, the CIPK23-CBL1/CBL9 complex activates the AKT1 potassium channel to enhance root K^+^ uptake, while CIPK8 interacts with CBL5 to improve salt tolerance (Ding et al., 2022). In potato, although the homolog of *CIPK8* has not yet been systematically characterized, the high conservation of the CBL-CIPK module across plant species suggests it may be involved in regulating potassium uptake and utilization. Recent advances have elucidated the post-translational regulation of CBL-CIPK modules in response to potassium availability. (Li et al., 2023) suggested that potassium nutrient status dynamically is associated with the phosphorylation status of CBL-CIPK-channel modules in Arabidopsis. The CBL1-CIPK23 complex activates the AKT1 potassium channel and the HAK5 high-affinity potassium transporter to enhance root K^+^ uptake under low-potassium conditions. CIPK8, while best known for its role in nitrate signaling, has also been implicated in potassium-related processes. In Arabidopsis, CIPK8 participates in CBL-CIPK networks that coordinate nitrate and potassium nutrition, and together with CIPK6 and CIPK16, mediates AKT1 activation. In potato, genome-wide analyses have identified 27 CIPK family members, but the specific roles of most members, including CIPK8, in potassium nutrition remain largely uncharacterized.

**Figure 1:**
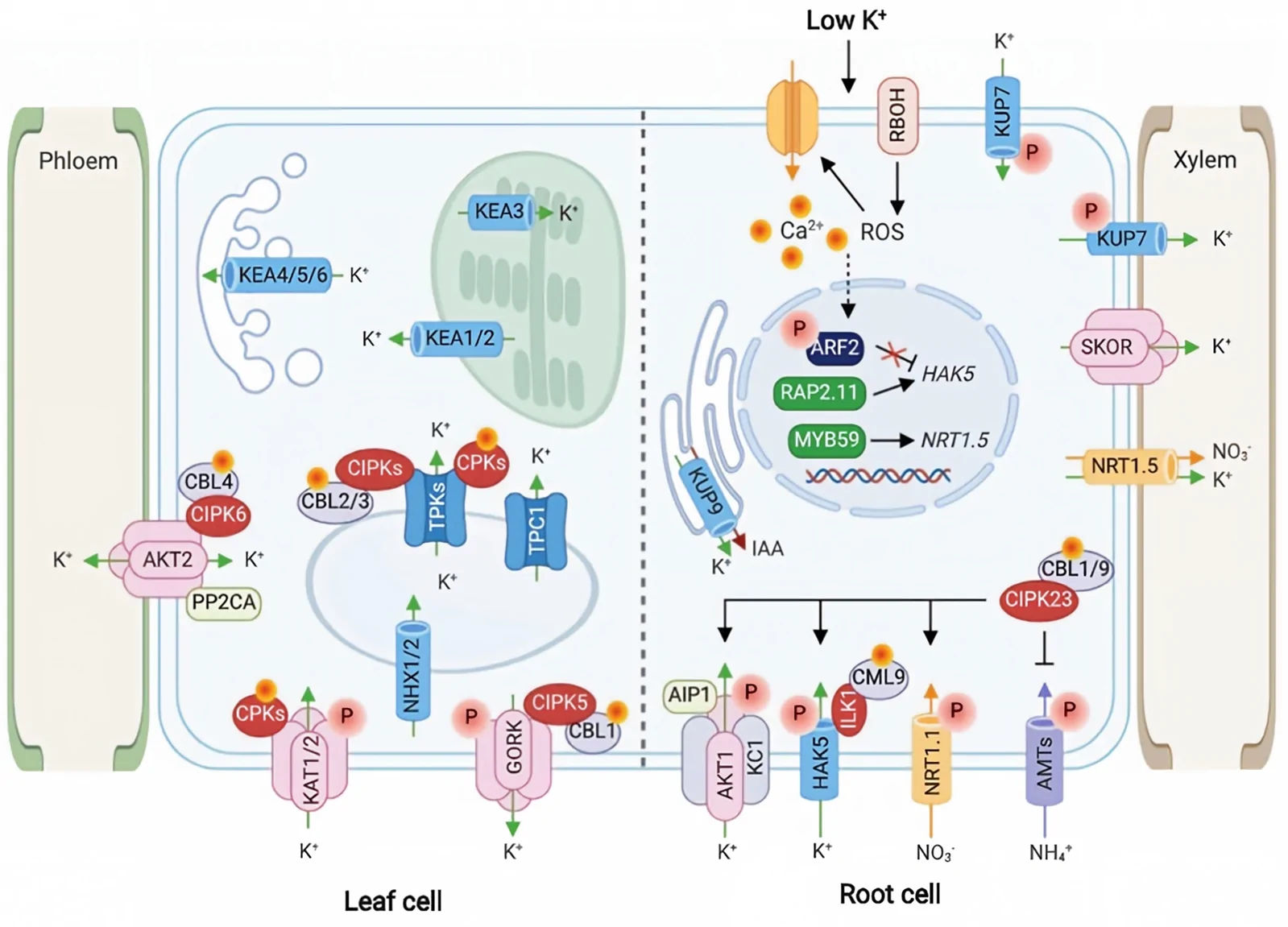
Schematic diagram of potassium uptake and transport regulatory network mediated by the CBL-CIPK signaling module^[25]^.

Among the 27 CIPK family members identified in the potato genome (Ma et al., 2021), CIPK8 was selected as the primary candidate based on three lines of evidence: phylogenetic conservation, functional precedent in other species, and our unpublished transcriptome co-expression data. First, the Arabidopsis ortholog AtCIPK8 is associated with the low-affinity phase of the primary nitrate response and participates in CBL–CIPK networks that coordinate nitrate and potassium nutrition (Hu et al., 2009); Second, closely related CIPK family members, including CIPK6, CIPK16, and CIPK23, activate AKT1 potassium channels in response to low potassium conditions (Lee et al., 2007; Li et al., 2023); Third, in our preliminary transcriptome co-expression network, CIPK8 emerged as a hub gene with high connectivity to multiple potassium transporters, potassium channels, and potassium-metabolism genes (**Figure 2**; unpublished data). This network centrality, together with its known roles in other species, made CIPK8 a compelling candidate for investigating potassium-regulated signaling in potato.

**Figure 2:**
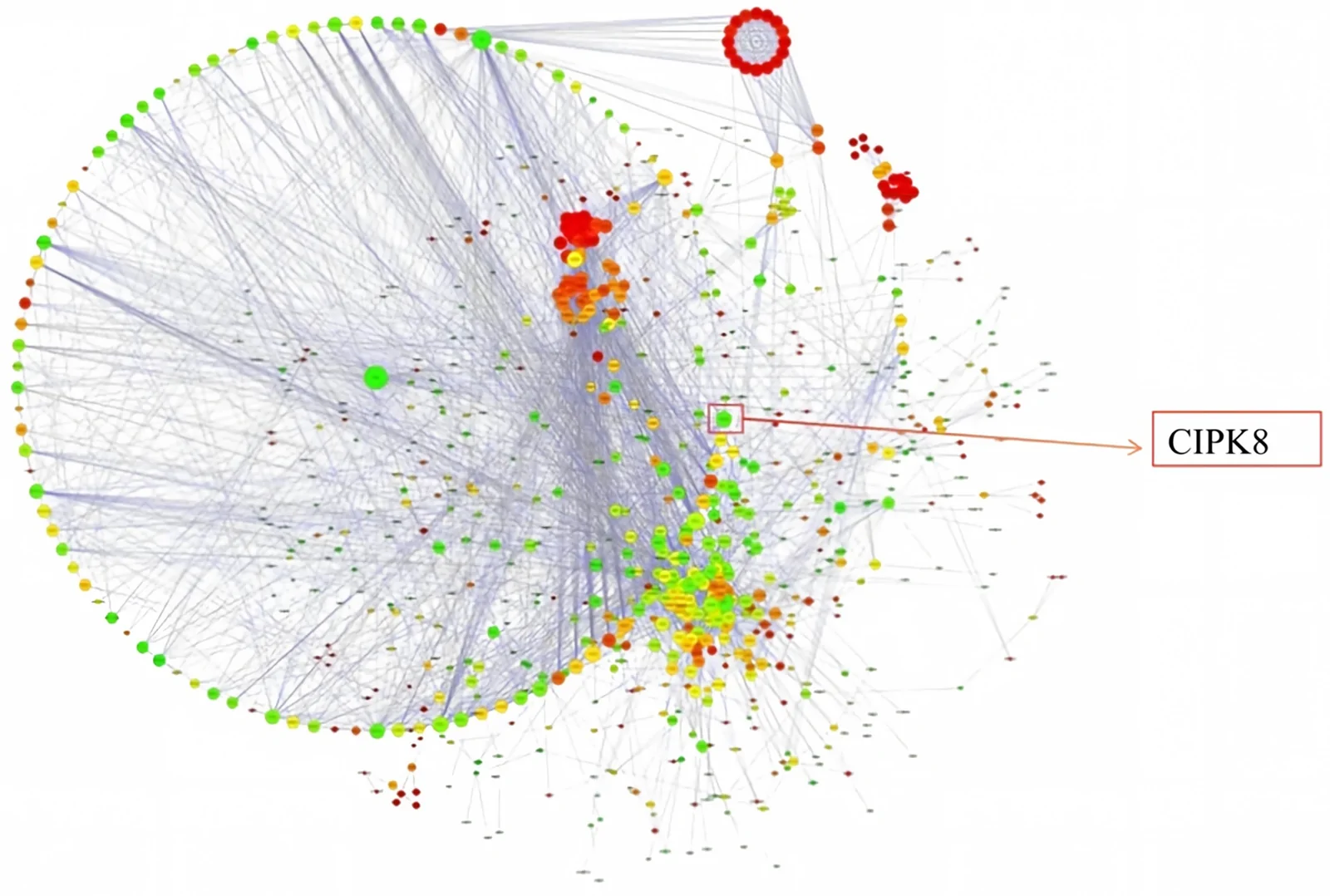
Gene regulatory network diagram.

While several potato CIPK family members have been characterized, most studies have focused on stress responses rather than potassium nutrition. For example, StCIPK10 has been shown to enhance drought and osmotic stress tolerance through ABA signaling pathways (Ma et al., 2021), and StCIPK15 functions in salt and heat stress responses (Zhu et al., 2025). In contrast, the role of CIPK8 in potato potassium nutrition has not been systematically investigated. Although CIPK23 is well-established as a positive regulator of AKT1-mediated potassium uptake in Arabidopsis, the functional specialization and divergence of CIPK8 from CIPK23 in potato remain unexplored. Our study represents the first attempt to characterize CIPK8 expression patterns in response to different potassium application rates in potato and to examine its subcellular localization.

Despite the recognized importance of potassium, the molecular mechanisms by which different potassium application rates affect potato yield—particularly through the *CIPK8*-associated pathway—remain largely unexplored. In this study, using the potato cultivar ‘V7’ as the experimental material, we applied five potassium application rates (K0, K120, K180, K240, and K300 kg·hm^-2^ K_2_O) over two consecutive years. We systematically investigated the effects of different potassium rates on potato yield and on the expression, protein abundance, and subcellular localization of *CIPK8*. Our aim was to characterize the expression pattern of CIPK8 in response to potassium application and to assess its potential association with potassium uptake and yield. This study provides a theoretical basis for efficient potassium use in potato production and offers practical guidance for optimizing potassium fertilizer management. This study aims to identify candidate genes responsive to potassium application and to characterize their expression patterns, rather than to establish a complete regulatory mechanism.

The new insights provided by this study include: (i) identification of 240 kg·hm^-2^ K_2_O as the optimal potassium rate for potato in the northern foothills of the Yinshan Mountains, based on two-year field trials; (ii) demonstration that CIPK8 gene and protein expression in potato exhibits a unimodal, concentration-dependent response to potassium supply, with peak expression at the tuber bulking stage (90 days); (iii) the first report of differential subcellular localization patterns between CIPK8 sequences (nucleus-enriched) and CIPK8 protein (nucleus, cytoplasm, and membrane) in potato, which may suggest multiple functional pools; and (iv) provision of a candidate gene for future functional studies on potassium use efficiency in potato. This study thus provides both a theoretical basis for efficient potassium use in potato production and practical guidance for optimizing potassium fertilizer management. We emphasize that this study aims to identify and characterize a candidate gene, not to establish a complete regulatory mechanism.

## 2 Materials and methods

### 2.1 Overview of the experimental site

The experiment was conducted at Wulanhua Town, Siziwang Banner, Ulanqab City, Inner Mongolia Autonomous Region, China. The previous crop was maize. The soil type at the experimental site was chestnut soil. The physical and chemical properties of the topsoil (0–20 cm) before sowing in 2024 and 2025 are shown in **Table 1**. The geographical coordinates range from 111°21′ to 111°34′ E and 41°34′ to 41°54′ N. The area has a temperate continental monsoon climate, with an average annual temperature of approximately 3.84 °C and an average annual precipitation of about 200 mm. The available potassium concentration of the topsoil (0–20 cm) before sowing was 90.00 mg·kg^-1^ in 2024 and 90.32 mg·kg^-1^ in 2025 (determined by ammonium acetate extraction–flame photometry). According to the National Second Soil Survey of China grading standards, this corresponds to a “moderate” level (50–100 mg·kg^-1^). The detailed soil physicochemical properties are shown in **Table 1**. Drip irrigation was applied to supplement the insufficient natural precipitation during the potato growing seasons. The irrigation system consisted of drip tapes laid along each ridge, with emitters spaced at 0.25 m. Irrigation was scheduled based on soil moisture monitoring and crop water requirements at different growth stages. All plots received identical irrigation regimes to ensure that the observed yield differences were attributable solely to potassium application rates.

**Table 1:** Physicochemical properties of experimental site soil.

Year	Organic matter (g/kg)	NH_4_^+^-N (mg/kg)	NO_3_^-^-N(mg/kg)	Available P(mg/kg)	Available K(mg/kg)	pH
2024	11.21	13.95	19.80	18.30	90.00	8.20
2025	11.82	13.62	20.10	17.90	90.32	8.00

### 2.2 Experimental design

The potato cultivar used in this experiment was ‘V7’. The fertilizers used were urea (N 46.0%) as nitrogen fertilizer, potassium sulfate (K_2_O 52.0%) as potassium fertilizer, and diammonium phosphate (N:P_2_O_5_ = 18.0:46.0) as compound fertilizer. The experimental design is shown in **Tables 2**, **3**. In 2024, five different potassium gradients were established: K_2_O 0 kg·hm^-2^ (K0), 120 kg·hm^-2^ (K120), 180 kg·hm^-2^ (K180), 240 kg·hm^-2^ (K240), and 300 kg·hm^-2^ (K300). After analyzing the 2024 yield data, the K120 treatment (120 kg·hm^-2^ K_2_O) was excluded from the 2025 repeated experiment because it consistently showed intermediate yields that were not significantly different from the K0 treatment in the first year (**Figure 3**), and the primary interest of the second-year experiment was to compare the optimal treatment (K240) with higher (K300) and moderate (K180) potassium rates to validate the unimodal yield response pattern. Thus, four treatments (K0, K180, K240, and K300) were selected for the repeated experiment in 2025. For each treatment, the application rates of nitrogen (N 250 kg·hm^-2^) and phosphorus (P_2_O_5–_200 kg·hm^-2^) were kept consistent. All fertilizers were mixed evenly and applied as a base fertilizer in a single application before sowing.

**Table 2:** Fertilizer application rates for each treatment in 2024.

Treatment	Fertilizer rate(kg·hm^-2^)		
	N	P_2_O_5_	K_2_O
K0	250	200	0
K120	250	200	120
K180	250	200	180
K240	250	200	240
K300	250	200	300

**Table 3:** Fertilizer application rates for each treatment in 2025.

Treatment	Fertilizer rate(kg·hm^-2^)		
	N	P_2_O_5_	K_2_O
K0	250	200	0
K180	250	200	180
K240	250	200	240
K300	250	200	300

**Figure 3:**
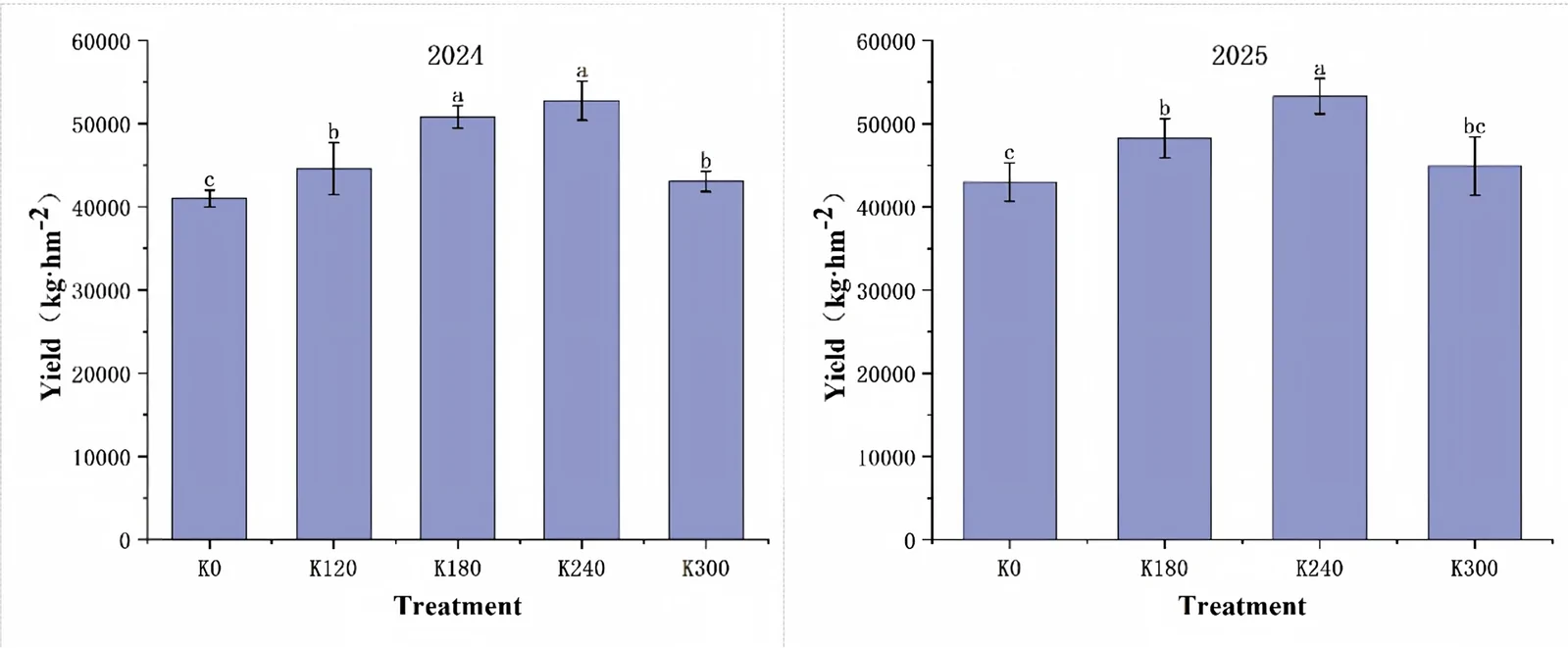
Effect of different potassium application rates on potato yield. Different lowercase letters in the figure indicate significant differences among treatments (P < 0.05).

A randomized complete block design was adopted, with three replicate plots per treatment. Each plot had an area of 65 m^2^, with a ridge width of 1.0 m, a total of five ridges, and a ridge length of 13 m. The plant spacing was 0.25 m. Two-meter-wide guard row was set up on both sides of each plot to prevent water and fertilizer cross-contamination. The experiment was sown in May and harvested in September in both years. The sampling time points at 50, 70, 90, and 110 days refer to days after planting (DAP), corresponding to the following potato growth stages: 50 DAP (vegetative growth stage), 70 DAP (tuber initiation stage), 90 DAP (tuber bulking stage), and 110 DAP (maturity/senescence stage), respectively.

### 2.3 Measurement items and methods

#### 2.3.1 Yield determination

At harvest, three sample plots of 1.0 m × 2.0 m were selected from the middle three ridges. All tubers within each sample plot were manually harvested, cleaned of soil, and diseased or rotten tubers were removed. The tubers were weighed, and the average value of the three replicates was calculated to determine the potato yield per unit area (kg·hm^-2^).

#### 2.3.2 Gene expression analysis and quantification

Based on transcriptome sequencing data of potato under different potassium application rates obtained in previous experiments in our laboratory, a gene co-expression network analysis method was used to screen key regulatory genes involved in potassium uptake and transport, and a gene regulatory network was constructed.

##### 2.3.2.1 RNA extraction

Total RNA was extracted from potato leaves and tubers using the YALEPIC Plant Total RNA Fast Isolation Kit-Plus (YALEPIC Biotechnology Co., Ltd., China) following the manufacturer’s instructions. RNA concentration and integrity were assessed by 1% agarose gel electrophoresis. The purified RNA was stored at −80 °C.

##### 2.3.2.2 Reverse transcription of RNA into cDNA

First-strand cDNA was synthesized using a reverse transcription kit according to the manufacturer’s protocol. The reaction conditions are shown in **Table 4**. The resulting cDNA was stored at −20 °C.

**Table 4:** Reverse transcription PCR reaction conditions.

Temperature condition(°C)	Reaction time(min)
37	15
50	5
98	5
4	∞

##### 2.3.2.3 Real-time quantitative PCR

Primers for CIPK8 and the reference gene EF1α were designed and synthesized by BGI Genomics Co., Ltd. (Shenzhen, China). Primer sequences are listed in **Table 5**. The cDNA was diluted 20-fold and used as a template. Conventional PCR was performed to verify primer specificity and determine optimal amplification conditions. The qPCR reaction mixture (**Table 6**) and thermal cycling conditions (**Table 7**) were used with EF1α as the internal control. Melting curve analysis was performed after the reaction, and gene expression levels were calculated using the 2^-ΔΔCt^ method. All qPCR reactions were performed using three independent biological replicates, each with three technical replicates. The mean values from biological replicates were used for statistical analysis.

**Table 5:** Primer sequences for RT-qPCR.

Name	Primer sequence(5'-3')
*CIPK8*	F:5'-GAAGCCTGAGAACCTACT-3' R:5'-ATAGTGATCCTCCTGTCC-3'
*EF1α*	F:5'-CCATTGGGTCGTTTTGCTGTG-3' R:5'-TTTGGCACCAGTTGGGTCCT-3'

**Table 6:** RT-qPCR reaction system.

Component	Volume(μL)
THUNDERBIRD SYBR qPCR Mix	10.0
Forward primer (F, 10 μmol/L)	0.2
Reverse primer (R, 10 μmol/L)	0.2
cDNA	1.0
ddH_2_O	8.6
Total	20.0

**Table 7:** RT-qPCR reaction conditions.

Step	Temperature(°C)	Time(s)	Number of cycles(次)
Initial denaturation	95	60	
Denaturation	95	15	
Annealing	Tm	20	
Extension	72	30	40
Melting stage	95	15	
Melting stage	60	60	
Melting stage	95	15	

#### 2.3.3 Interpretation of this circular CIPK phylogenetic tree

The potato *CIPK8* gene was identified through BLASTP search against the Solanum tuberosum genome v4.03 (PGSC_DM_v4.03) using the Arabidopsis thaliana CIPK8 protein sequence (AT4G24400) as a query. The potato gene corresponding to the CIPK8 ortholog was designated as PGSC0003DMG400033335 (transcript ID: PGSC0003DMT400083474; protein ID: PGSC0003DMP400055981), located on chromosome 11 (ST4.03ch11: 37, 703, 624–37, 708, 616, negative strand). Full-length protein sequence alignment revealed that *StCIPK8* shares approximately 46% amino acid identity and 63% similarity with *AtCIPK8* (AT4G24400), confirming it as the putative ortholog of *AtCIPK8* in potato. This gene was previously identified as a member of the CIPK family in a genome-wide phylogenetic analysis of potato *CIPK* genes (Ma et al., 2021), in which the 27 potato CIPK family members were classified into five groups. Consistent with that classification, *StCIPK8* clusters within the *CIPK8* clade (**Supplementary Figure S1**). The gene-specific primers for *CIPK8* used in qRT-PCR were designed based on the coding sequence of this gene (**Table 5**).

#### 2.3.4 Transcriptome sequencing, data processing, and co-expression network construction

##### RNA extraction, library preparation, and sequencing

Total RNA was extracted from potato leaf samples using TRIzol^®^ Reagent (Invitrogen, CA, USA) following the manufacturer’s protocol. RNA purity and concentration were assessed using a NanoDrop 2000 Spectrophotometer (Thermo Fisher Scientific, MA, USA), and RNA integrity was evaluated using the Agilent 2100 Bioanalyzer with the RNA Nano 6000 Assay Kit (Agilent Technologies, CA, USA). Only samples with RNA integrity number (RIN) ≥ 8.0 were used for subsequent library construction. A total of 15 RNA samples were sequenced, including three biological replicates for each of the five potassium treatments (K0, K120, K180, K240, and K300) from the 2024 field experiment.

Sequencing libraries were generated using the NEBNext^®^ Ultra™ RNA Library Prep Kit for Illumina^®^ (NEB, USA) following the manufacturer’s recommendations, and index codes were added to multiplex each sample. Library quality was assessed using the Qubit^®^ 2.0 Fluorometer for initial quantification and the Agilent 2100 Bioanalyzer for insert size detection. The effective library concentration (>2 nM) was determined by quantitative PCR (qPCR). The indexed libraries were pooled and sequenced on the Illumina NovaSeq 6000 platform, generating 50 bp single-end raw reads.

##### Read processing, mapping, and quantification

Raw reads were filtered to remove low-quality reads (those with > 50% bases having a Qphred score ≤ 5), reads containing more than 10% ambiguous bases (N), and reads with adapter contamination using Trimmomatic (v0.39). The resulting clean reads were mapped to the Solanum tuberosum reference genome (PGSC_DM_v4.03) using HISAT2 (v2.2.1) with default parameters. Gene expression levels (transcripts per million, TPM) for each sample were quantified using StringTie (v2.1.4) and featureCounts (v2.0.1). Only genes with TPM ≥ 1 in at least 50% of the samples were retained for downstream analysis.

##### Differential expression analysis

Differential expression analysis between potassium treatment groups was performed using the DESeq2 R package (v1.36.0). Genes with an adjusted P-value < 0.05 and |log_2_ (fold change) | ≥ 1 were considered significantly differentially expressed. These differentially expressed genes were used as input for the subsequent co-expression network analysis.

##### Co-expression network construction and hub gene identification

To identify key regulatory genes responsive to potassium application, a weighted gene co-expression network was constructed using the WGCNA R package (v1.71). Based on the scale-free topology criterion, a soft-thresholding power of β = 6 (R^²^ > 0.85) was selected. The adjacency matrix was transformed into a topological overlap matrix (TOM), and modules were identified using the dynamic tree-cut algorithm with a minimum module size of 30 genes. Module eigengenes were calculated, and modules significantly correlated with potassium treatment were selected. Hub genes were defined as those with module membership (MM) > 0.8 and gene significance (GS) > 0.5 for the trait of interest. The co-expression network was visualized using Cytoscape (v3.9.1).

#### 2.3.5 fluorescence *in situ* hybridization assay

Tissue sections were prepared, deparaffinized, rehydrated, and then hybridized with a FAM-labeled CIPK8 probe. Prehybridization was performed at 37 °C for 30 min using a prehybridization solution (blocking solution : prehybridization buffer = 1:99). Hybridization was carried out overnight at 37 °C in the dark with the probe mixture (2.5 μL probe stock in 100 μL pre-warmed hybridization solution). After hybridization, sections were washed under dark conditions at 42 °C: three times with Wash Solution I (5 min each), once with Wash Solution II (5 min), once with Wash Solution III (5 min), and finally rinsed with PBS at room temperature for 5 min. Nuclei were counterstained with DAPI (5 min), and sections were observed under a fluorescence microscope. Images were captured and saved. The FISH experiment was performed three times independently with similar results; representative images are shown. Autofluorescence was not specifically quenched, the low green-channel background may be due to the short exposure time and the use of a FAM-labeled probe with minimal free dye; however, the green-channel background was consistently low across sections, and DAPI and FAM channels were acquired separately to minimize bleed-through. No-probe, sense-probe, and RNase/DNase controls were not performed in this study. Therefore, the nuclear FISH signal should be interpreted as indicating the presence of CIPK8 sequences in the nucleus, which may include genomic DNA or nascent transcripts, rather than being definitively assigned to mature mRNA.

#### 2.3.6 Western blot assay for protein expression levels

Total protein was extracted from potato leaves sampled at the same time points as those for qPCR. For K0, K240, and K300 treatments, potato leaves with consistent growth status were selected. Fully expanded functional leaves were rinsed with ultrapure water, blotted dry with sterile filter paper, and 0.5 g of sample was cut and placed into a pre-chilled mortar. Sufficient liquid nitrogen was added and the tissue was rapidly ground into a fine powder to avoid protein degradation due to thawing. The powder was then mixed with pre-cooled protein extraction buffer containing protease inhibitors, incubated on ice for 30 min, and centrifuged at 12, 000 rpm for 20 min at 4 °C. The supernatant was collected, and protein concentration was determined using a BCA kit. Samples were mixed with 5× loading buffer (4:1 v/v), heated at 100 °C for 10 min, and subjected to SDS-PAGE. After electrophoresis, proteins were transferred to a PVDF membrane (activated with methanol for 30 min) at 100 V for 1-1.5 h at 4 °C. The membrane was blocked with 5% non-fat milk in TBST for 1 h at room temperature, then incubated with primary antibody overnight at 4 °C, followed by HRP- or fluorescence-labeled secondary antibody for 1.5 h at room temperature. Protein bands were visualized using ECL chemiluminescence, and band intensity was quantified using ImageJ with GAPDH as the loading control. Western blot analysis was repeated with three independent biological replicates, and representative images are shown. Band intensity quantification was performed on all three replicates.

#### 2.3.7 Subcellular localization

Tissue sections were deparaffinized, rehydrated, and treated with 3% H_2_O_2_ for 10 min. Antigen retrieval was performed in a pH 9.0 EDTA buffer using a microwave oven (medium power 8 min, pause 5 min, low power 5 min). After cooling and washing, sections were blocked with 5% BSA at 37 °C for 30 min, then incubated with primary antibody against CIPK8 (dilutions 1:50, 1:100, 1:200) overnight at 4 °C. After washing, sections were incubated with species-matched secondary antibody at 37 °C for 30 min, followed by DAPI staining for 10 min at room temperature in the dark. Mounted sections were observed under a confocal microscope, and images were captured. Subcellular localization was examined in at least three independent experiments, and representative confocal images are presented. All images were acquired with identical laser settings across samples. No-primary-antibody and peptide-competition controls were not performed in this study. Therefore, although three primary antibody dilutions (1:50, 1:100, 1:200) produced consistent localization patterns, the possibility of non-specific antibody binding cannot be fully excluded, and the localization results should be considered preliminary.

#### 2.3.8 Determination of plant potassium concentration and potassium use efficiency

At harvest, representative plant samples (leaves, stems, and tubers) were collected from each plot. The samples were oven-dried at 105 °C for 30 min, then at 70 °C to constant weight, and ground to pass through a 0.5-mm sieve. Potassium concentration was determined by H_2_SO_4_-H_2_O_2_ digestion followed by flame photometry (Sherwood M410, UK). Plant potassium accumulation (kg K·ha^-1^) was calculated as:


K accumulation = (shoot dry weight × shoot K concentration) + (tuber dry weight × tuber K concentration)

Potassium fertilizer agronomic efficiency (AE_K, kg·kg^-1^) was calculated as: $\text{AE}\_\text{K }=\;\left(\text{yield}\_\text{K }-\text{ yield}\_\text{control}\right)/{\text{K}}_{2}\text{O application rate}$

Potassium fertilizer partial factor productivity (PFP_K, kg·kg^-1^) was calculated as: $\text{PFP}\_\text{K }=\text{ yield}\_\text{K}/{\text{K}}_{2}\text{O application rate}$

Potassium utilization efficiency (KUE, kg tuber yield per kg K accumulation) was calculated as: KUE = tuber yield/total plant K accumulation

### 2.4 Data statistics and analysis methods

Data were analyzed using linear mixed-effects models implemented in the R package lme4 (v1.1-34). For yield data, the model was specified as: Yield ~ Year + K_rate + Year: K_rate + (1|Block), where Year, K rate, and their interaction were fixed effects, and Block was a random intercept. For gene and protein expression data measured at multiple time points (50, 70, 90, and 110 DAP), the model was specified as: Expression ~ K_rate + DAP + K_rate: DAP + (1|Block/Plot), where K rate and DAP were fixed effects, and Plot nested within Block was a random intercept; repeated measures within plot were modeled with an autoregressive (AR1) covariance structure. Tukey’s HSD test was used for *post-hoc* multiple comparisons at a significance level of P < 0.05. SPSS 23.0 was used only for descriptive statistics and data visualization; all inferential statistics were performed in R. To determine the optimal potassium application rate, a quadratic regression model (y = a + bx + cx^²^) was fitted to the yield data from each year using the nls function in R. In addition to the quadratic model, a linear-plateau model was fitted to the yield data to better capture the potential plateau response at higher K rates. The model was expressed as:


Y=a+b×min(K, C)+ϵ

where *Y* is the predicted yield, *K* is the K_2_O application rate, *a* is the intercept, *b* is the slope of the linear increase phase, and *C* is the critical K rate (breakpoint) at which yield reaches a plateau. Model fitting was performed using the segmented package in R (v4.2.0). Goodness-of-fit was compared between the quadratic and linear-plateau models using R² and Akaike Information Criterion (AIC).

Graphs were generated using Origin 2021 (OriginLab, USA) and ggplot2 (v3.4.0). All data are presented as mean ± standard deviation (SD). In all figures, data are presented as mean ± SD. Different lowercase letters above the bars indicate significant differences among treatments at *P* < 0.05 (Tukey’s HSD test).

## 3 Results

### 3.1 Yield

Potassium application rate significantly affected potato tuber yield in both experimental years (*P* < 0.001). The linear mixed-effects model included Year, K rate, and their interaction as fixed effects and block as a random intercept. The model revealed a highly significant main effect of K rate (*P* < 0.001), while the Year × K rate interaction was not significant (*P* = 0.31), indicating that the yield response pattern was consistent across the two seasons.

In 2024, yield initially increased with escalating K application rates, peaking at K240 (52, 800 kg·hm^-2^), which was significantly higher than K0, K120, and K300, but comparable to K180. The K300 treatment showed a marked yield reduction compared with K240, with yields dropping to approximately 43, 000 kg·hm^-2^, only marginally higher than the K0 control (41, 000 kg·hm^-2^). In 2025, the same unimodal pattern was observed, with K240 again producing the highest yield (53, 300 kg·hm^-2^), significantly surpassing K0, K180, and K300 (*P* < 0.05) (**Figure 3**).

To further characterize the yield response, a quadratic regression model was fitted to the yield data from each year. The relationship between K rate and yield was described by the equations y = -0.295x^²^ + 106.96x + 39948 (R^²^ = 0.522) for 2024 and y = -0.269x^²^ + 93.09x + 42722 (R^²^ = 0.504) for 2025. The theoretical optimum K application rates derived from these equations were 181.2 kg·hm^-2^ (predicted maximum yield: 49, 639 kg·hm^-2^) in 2024 and 172.9 kg·hm^-2^ (predicted maximum yield: 50, 763 kg·hm^-2^) in 2025.

However, because the yield response appeared to reach a plateau rather than following a symmetric rise-and-fall pattern, we also fitted a linear-plateau model (**Figure 4**). This model estimated the critical K rate (breakpoint) at which yield reaches a plateau as 180.0 kg·hm^-2^, with a predicted plateau yield of 48, 579 kg·hm^-2^. The linear-plateau model showed improved goodness-of-fit (R^²^ = 0.61) compared with the quadratic model (R^²^ = 0.52), and the Akaike Information Criterion was lower, indicating that it provided a more parsimonious description of the yield response. Importantly, the predicted plateau yield was closer to the observed yields at K240 (52, 800 and 53, 300 kg·hm^-2^) than the quadratic predictions (49, 639 and 50, 763 kg·hm^-2^).

**Figure 4:**
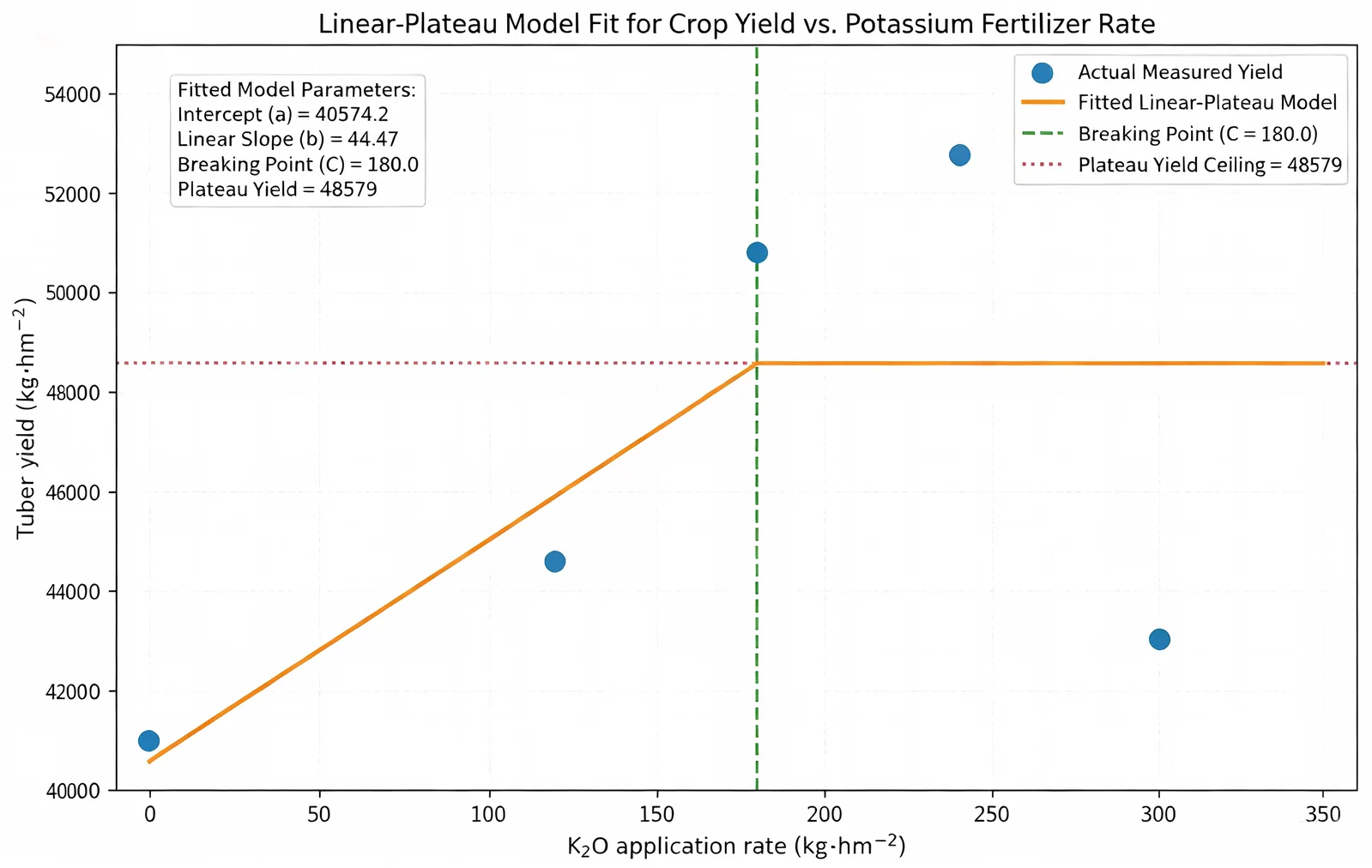
Linear-plateau model fit for potato tuber yield in response to potassium fertilizer application rate (2024 data; the 2025 season showed a similar pattern). The solid line represents the fitted model. The vertical dashed line indicates the estimated breakpoint at 180.0 kg·hm^-^², and the horizontal dashed line indicates the estimated plateau yield of 48,579 kg·hm^-^². The shaded area represents the 95% confidence interval. R² = 0.61. Data points are treatment means ± SD (n=3). This model provided a better description of the yield response than the quadratic model, with the breakpoint falling between the K180 and K240 treatments.

The linear-plateau analysis suggests that the breakpoint falls between K180 and K240. While K180 approached the plateau threshold in 2024, K240 consistently outperformed K180 in 2025, indicating that a higher rate within the plateau range may be needed to buffer against year-to-year environmental variability. The consistent decline in yield at K300 across both years confirms that excessive potassium application (>240 kg·hm^-2^) provides no additional yield benefit and may even reduce productivity.

### 3.2 Screening of key genes for potassium uptake in potato under different potassium application rates

As shown in **Figure 2**, within the functional modules related to potassium nutrition response, the *CIPK8* gene exhibited a relatively high core regulatory status. This gene showed high levels of both connectivity and centrality in the co-expression network, serving as a key hub node connecting the potassium uptake functional module with other metabolic pathways. It also displayed close co-expression relationships with multiple potassium transporters, potassium channel proteins, and other genes related to potassium metabolism. These findings suggest that *CIPK8* may act as an upstream core regulatory factor, playing an important hub role in potassium uptake, transport, and homeostasis maintenance in potato, thereby providing a key candidate gene for subsequent functional validation.

### 3.3 Effect of different potassium application rates on *CIPK8* gene expression in potato

For CIPK8 gene expression, repeated-measures linear mixed-effects models were fitted with K rate, DAP, and their interaction as fixed effects and plot nested within block as a random intercept (AR1 covariance structure). The same model structure was used for protein expression in Section 3.5. The qRT-PCR validation results from 2024 and 2025 (**Figure 5**) showed that the expression level of the *CIPK8* gene in potato leaves exhibited a unimodal curve over the growth period, first increasing and then decreasing. The highest expression level was observed at 90 DAP under all treatments, indicating a high degree of coordination between gene expression and plant physiological function. At 50 DAP, plant metabolism was relatively low, and *CIPK8* expression was low; compared with the K0 treatment, the K240 treatment increased expression by approximately 57.29% and 62.94%, respectively, but there were no significant differences among the K180, K240, and K300 treatments, At 70 DAP, *CIPK8* expression gradually increased; compared with the K0 treatment, the K240 treatment increased expression by approximately 43.33% and 40.02%, respectively. At 90 DAP, *CIPK8* expression reached its peak across the entire growth period, and the expression level under the K240 treatment was significantly higher than that under all other treatments, with increases of up to 57.00% and 53.81% compared with the K0 treatment, representing the largest increase over the whole growth period. Compared with the K120, K180, and K300 treatments, the K240 treatment increased expression by approximately 12.61%, 7.53%, and 6.31%, respectively, and the K300 treatment showed a slight concentration-dependent inhibitory effect. At 110 DAP, leaf physiological function declined, and *CIPK8* expression generally decreased; nevertheless, the K240 treatment still significantly increased expression by approximately 38.70% and 37.16% compared with the K0 treatment, indicating that potassium application could maintain relatively high *CIPK8* expression during the senescence stage, thereby delaying leaf functional decline.

**Figure 5:**
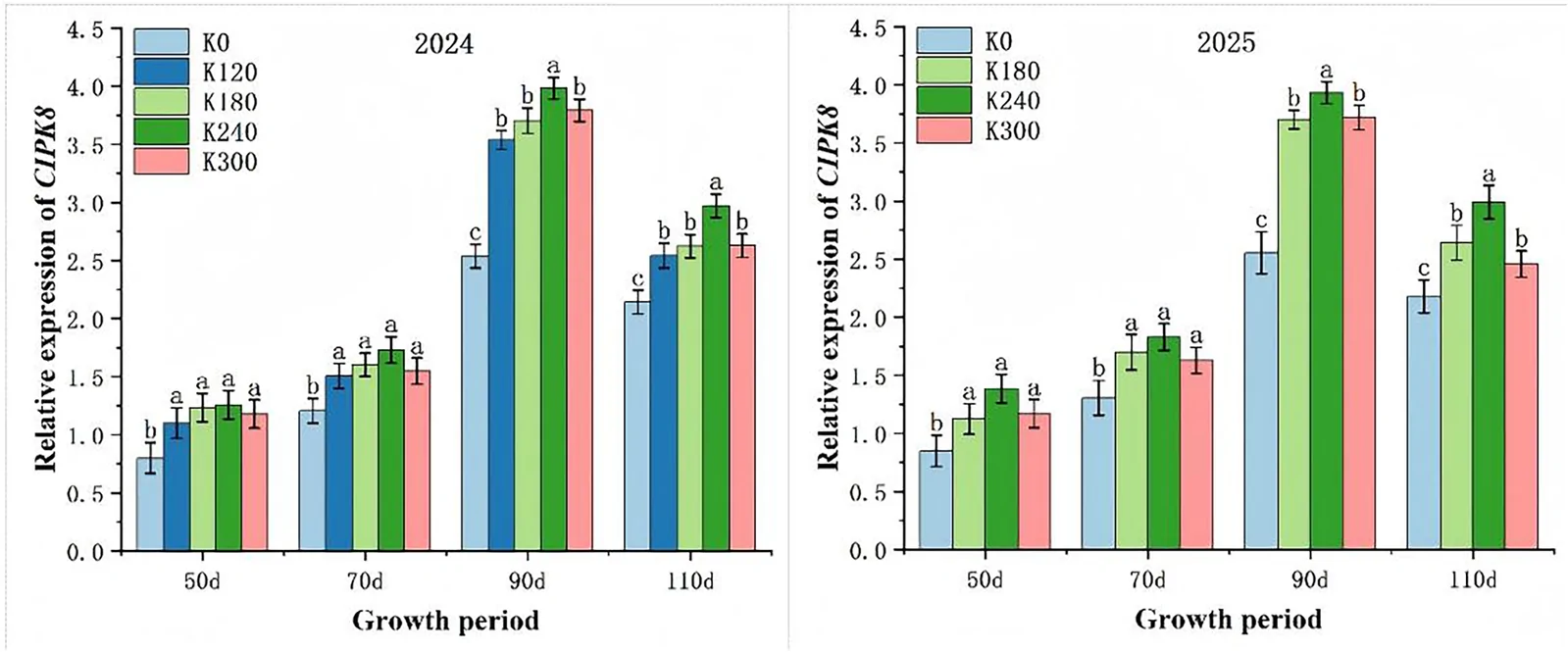
Effect of different potassium application rates on *CIPK8* gene expression in potato. Relative expression of the CIPK8 gene in potato plants under different potassium (K) treatments across various growth stages in 2024 and 2025. The x-axis represents the growth period (50, 70, 90, and 110 days), and the y-axis represents the relative expression of CIPK8. Different colored bars represent different K fertilization levels. Error bars indicate the standard deviation (SD), and different lowercase letters (a–c) above the bars denote significant differences among the K treatments within the same growth stage (P < 0.05).

The results showed that the expression level of the *CIPK8* gene under the K240 treatment was highly coordinated with the physiological patterns of the leaf antioxidant system and starch synthesis. At 90 days, a key period for *CIPK8* response to potassium nutrition, the expression level under the K240 treatment increased by more than approximately 50% compared with the K0 treatment, representing the largest increase over the whole growth period.

### 3.4 Fluorescence *in situ* hybridization analysis of the *CIPK8* gene

According to the results shown in **Figure 6**, the left panel shows blue fluorescence from DAPI staining, marking the cellular contours and nuclear regions of tissue cells, clearly presenting the cellular structural background of potato tissues; the middle panel shows green fluorescence from the specific hybridization signal of the *CIPK8* gene, displaying the expression distribution of the target gene alone; and the right panel shows the merged image of DAPI and FAM signals, intuitively verifying the localization characteristics of the signal. The FISH results showed that the green fluorescence signal (FAM) of the CIPK8 probe was detected in both the nucleus and the cytoplasm, with partial overlap with the DAPI-stained nuclear region. Background fluorescence was low, but because no-probe, sense-probe, and RNase/DNase controls were not performed, this signal may represent hybridization to either genomic DNA or nascent transcripts in the nucleus. Thus, the signal indicates the presence of CIPK8 sequences in the nucleus, but its assignment to mature cytoplasmic mRNA requires further validation with RNA-FISH controls. Nevertheless, the observed localization pattern is broadly consistent with the expected function of CIPK family members as signaling components involved in calcium signal transduction.

**Figure 6:**
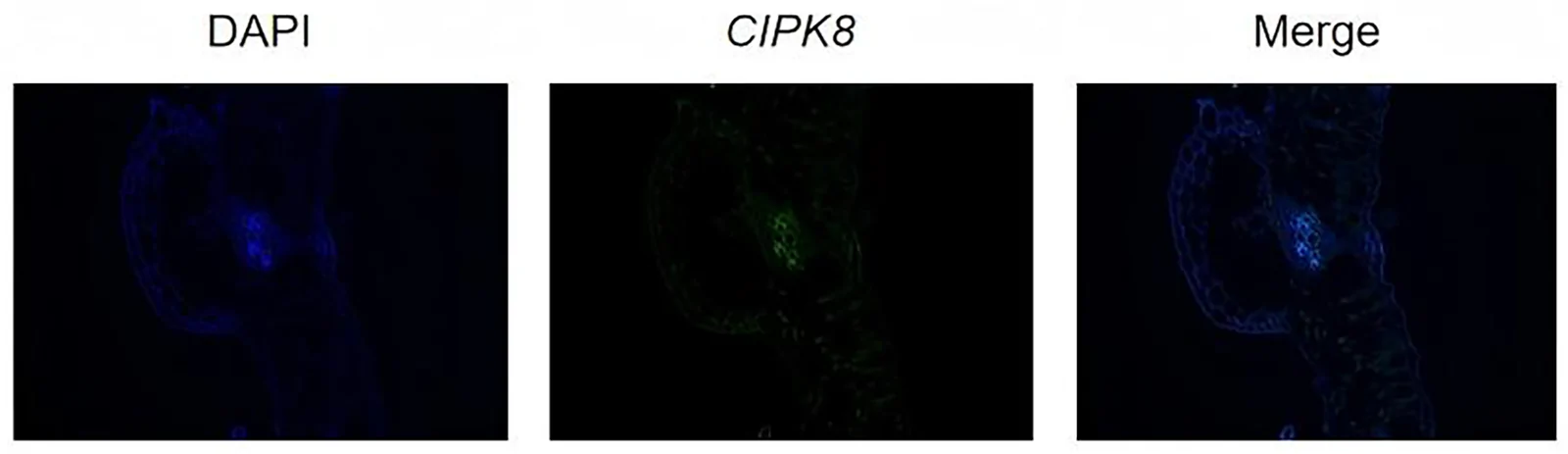
FISH results of *CIPK8* gene under different potassium application treatments. Fluorescence *in situ* hybridization (FISH) analysis of the *CIPK8* gene in potato. Left: DAPI staining for cell contours and nuclei (blue fluorescence); Middle: FAM-labeled specific hybridization signal of the *CIPK8* gene (green fluorescence); Right: Merged image. Scale bar = 200 μm.

### 3.5 Effect of different potassium application rates on CIPK8 protein expression in potato

The results (**Figure 7**) showed that CIPK8 protein expression peaked at 90 DAP. The K240 treatment significantly upregulated CIPK8 protein expression, with expression levels significantly higher than those under the K0 and K300 treatments throughout the entire growth period. The highest increase relative to the K0 treatment was observed at 90 days. These results indicate that the K240 treatment is associated with upregulation of CIPK8 expression and may contribute to potassium uptake capacity. Under the K300 treatment, CIPK8 expression was significantly lower than that under the K240 treatment, failing to further improve the potassium nutrition effect. The expression pattern of CIPK8 protein was consistent with plant potassium accumulation and yield changes, providing molecular support for K240 as the optimal potassium application level. However, these data are correlative and do not establish a causal role for CIPK8.

**Figure 7:**
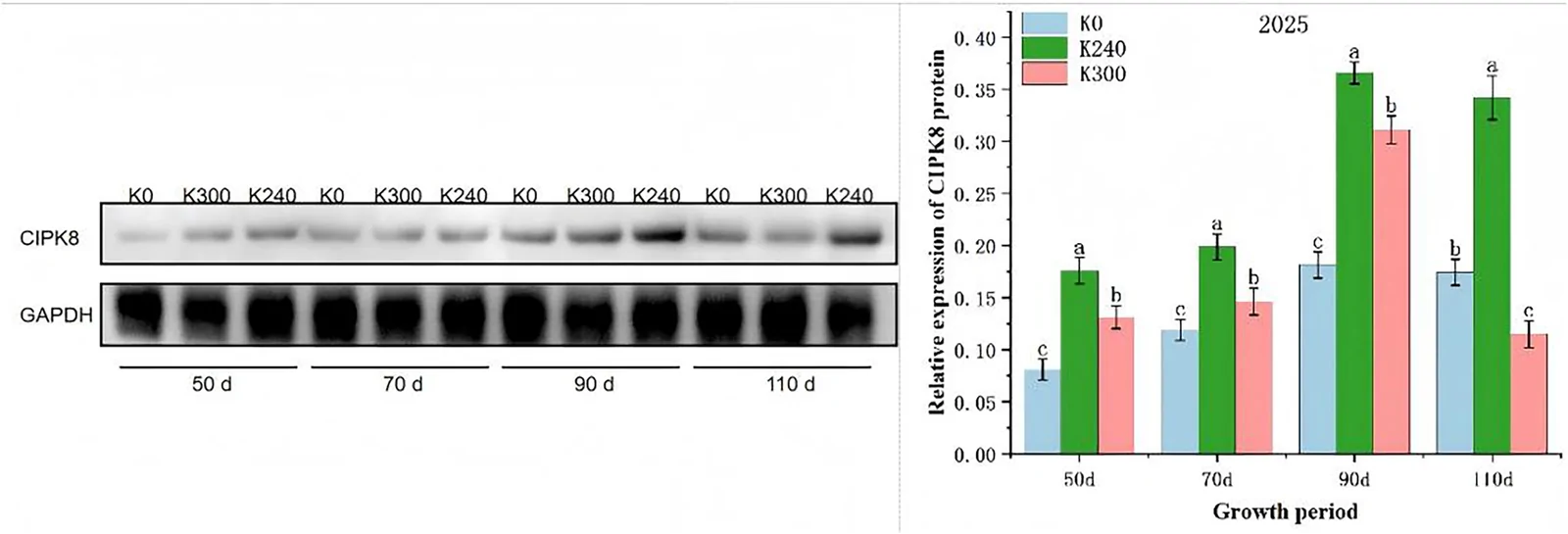
Effect of different potassium application rates on CIPK8 protein expression in potato. CIPK8 protein expression levels under different potassium treatments at 50, 70, 90, and 110 days after planting (DAP). GAPDH was used as the loading control. Band intensity was quantified from three independent biological replicates. Different lowercase letters indicate significant differences among treatments at each time point (*P* < 0.05, Tukey’s HSD).

### 3.6 Subcellular localization analysis of CIPK8 protein

The subcellular distribution characteristics of the CIPK8 protein in potato were systematically detected by immunofluorescence labeling technology. Three primary antibody dilution gradients (1:50, 1:100, and 1:200) were set up in the experiment, and the results are shown in **Figure 8**. In the figure, blue fluorescence represents the nuclear regions stained with DAPI, and purple fluorescence represents the specific immunofluorescence signal of the CIPK8 protein. In vascular bundle cells of the leaf main vein (**Figure 8**), the merged image showed that the fluorescence signal of the CIPK8 protein was observed in the nuclear region of the vascular bundles, partially overlapping with the DAPI-stained nuclear positions. Only extremely weak background fluorescence was observed in other subcellular structures such as the cytoplasm and cell membrane, with no obvious specific signal aggregation. However, because no-primary-antibody and peptide-competition controls were not performed, the possibility of non-specific binding cannot be fully excluded.

**Figure 8:**
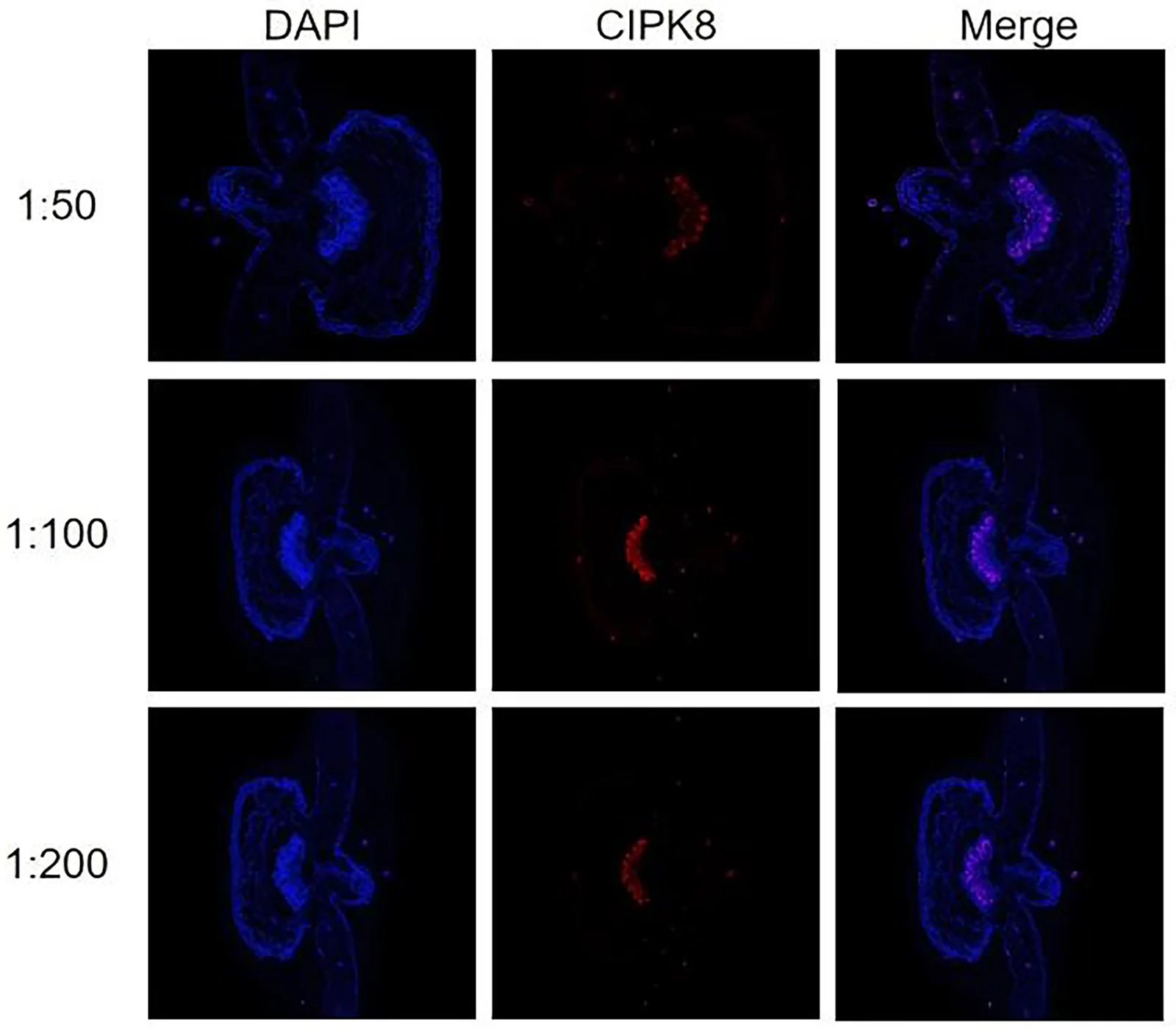
Results of antibody screening for subcellular localization of CIPK8 protein in potato. Subcellular localization of CIPK8 protein in potato under different primary antibody dilution gradients. From top to bottom: 1:50, 1:100, and 1:200, respectively. From left to right: DAPI nuclear staining (blue), CIPK8 protein immunofluorescence signal (red), and merged image.

Further analysis of the signal characteristics at different dilution gradients showed that the nuclear localization of the CIPK8 protein was stable and consistent across all primary antibody dilution gradients tested. Among them, the 1:100 dilution group exhibited moderate fluorescence signal intensity with minimal background interference, and the nuclear localization signal was clear and sharp, making it the optimal working concentration for this antibody to detect the CIPK8 protein. The 1:50 high-concentration group showed slight non-specific background fluorescence, while the 1:200 low-concentration group showed a slight attenuation of signal intensity. However, neither of these two groups altered the nuclear localization pattern of the CIPK8 protein, and the core conclusion remained consistent. These results should be interpreted as preliminary because antibody specificity was not independently validated by no-primary or peptide-competition controls.

In summary, the immunofluorescence localization results indicated that the CIPK8 protein in potato was observed mainly in the nucleus under the tested conditions. In mesophyll cells (**Figure 9**), the immunofluorescence results showed that CIPK8 (red fluorescence signal) was observed in the cell membrane and cytoplasm, with partial signals also present in the nuclear region, suggesting a multi-compartment distribution in fixed tissues involving the membrane, cytoplasm, and nucleus. The apparent difference in CIPK8 localization between vascular bundle cells (**Figure 8**, predominantly nuclear) and mesophyll cells (**Figure 9**, nucleus, cytoplasm, and membrane) may reflect cell-type-specific distribution patterns rather than a contradiction. Different cell types may exhibit distinct subcellular localization of *CIPK8* protein depending on their physiological functions.

**Figure 9:**
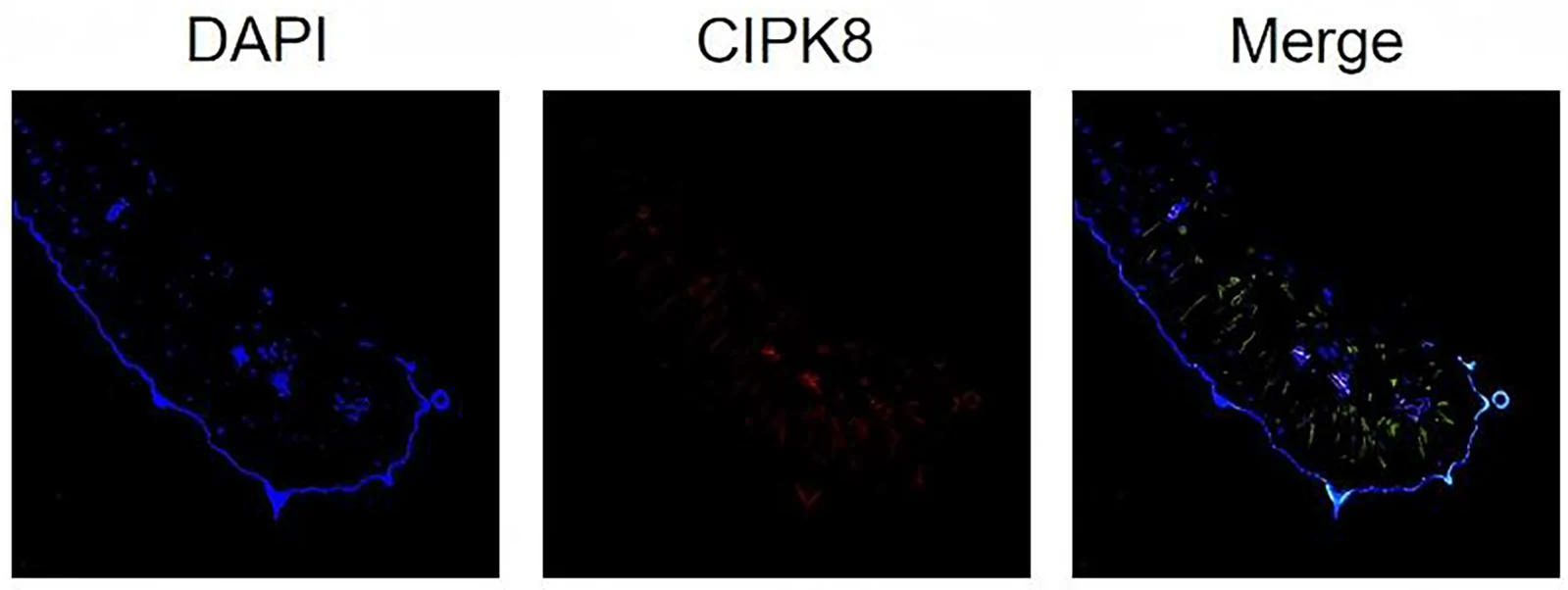
Subcellular localization analysis of CIPK8 protein in potato. Immunofluorescence localization of CIPK8 protein in mesophyll cells of potato leaves. Blue fluorescence (DAPI) indicates nuclei; red fluorescence indicates CIPK8 protein signal. The merged image shows CIPK8 protein distributed in the nucleus, cytoplasm, and plasma membrane in mesophyll cells. Three independent experiments were performed with similar results; representative images are shown. The apparent difference in subcellular localization between **Figures 7**, **Figure 8** reflects cell-type-specific distribution patterns rather than a contradiction: **Figure 7** shows CIPK8 localization in vascular bundle cells (predominantly nuclear), while **Figure 8** shows CIPK8 localization in mesophyll cells (nucleus, cytoplasm, and plasma membrane).

### 3.7 Potassium concentration, accumulation, and use efficiency

#### 3.7.1 Potassium concentration in different organs

The experimental results from both 2024 and 2025 (**Figures 10**, **11**) indicated that potassium (K) application rates significantly affected the K concentration in various potato organs (*P* < 0.05). With increasing K application levels, the K concentration in all organs (roots, stems, leaves, and tubers) showed an upward trend, with significant differences among treatments. The K300 treatment consistently resulted in the highest K concentration across all organs, while the K0 treatment yielded the lowest. The inclusion of the K120 treatment in the 2024 experiment further suggested the gradient increase in K concentration corresponding to K application rates.

**Figure 10:**
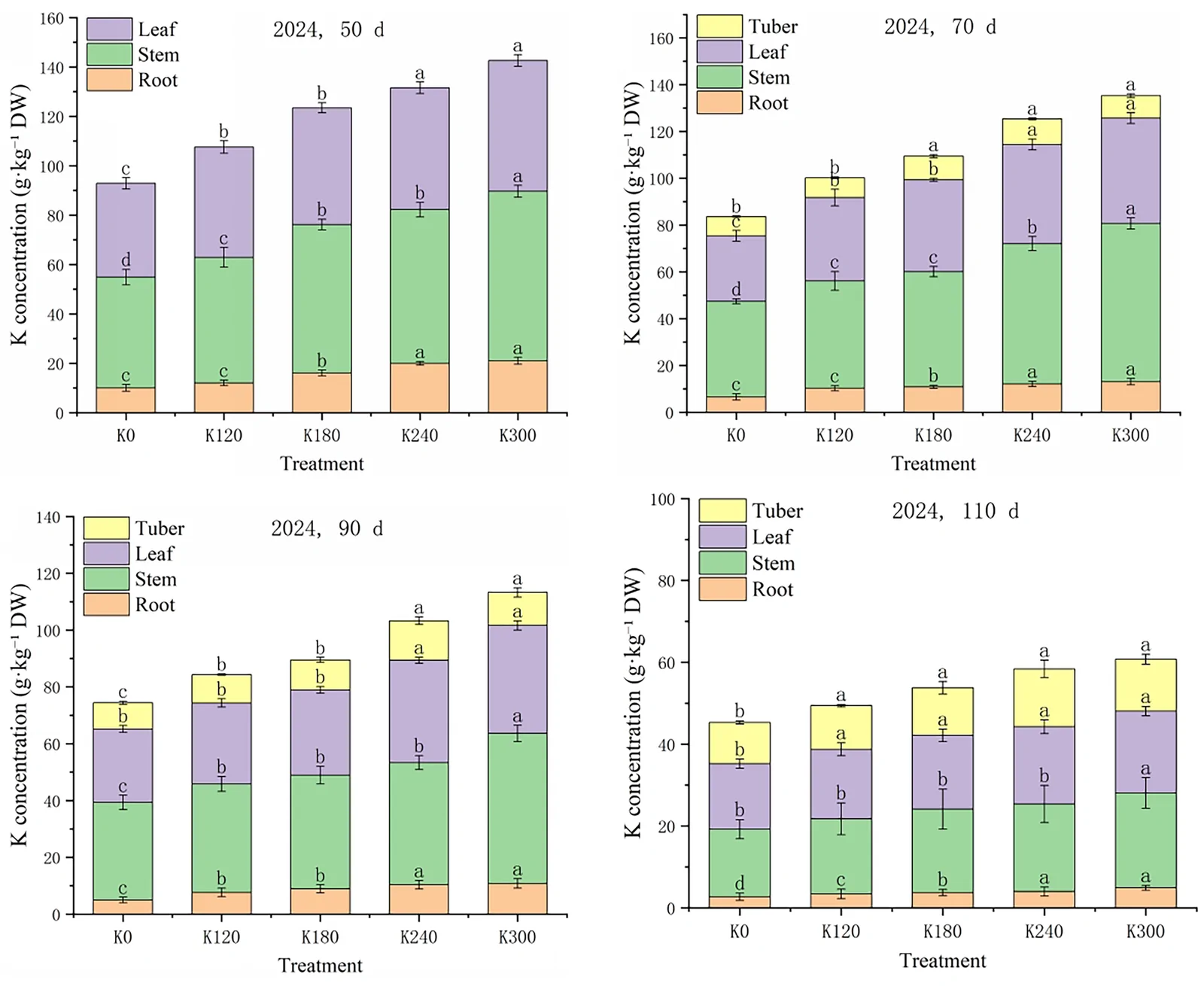
Effects of different potassium application rates on potassium concentration (g kg^-1^ DW) in different organs (roots, stems, leaves, and tubers) of potato in 2024. Data are expressed as mean ± SD (n=3). Different lowercase letters indicate significant differences among treatments at the same growth stage (P < 0.05, Tukey’s HSD test). DW, dry weight.

**Figure 11:**
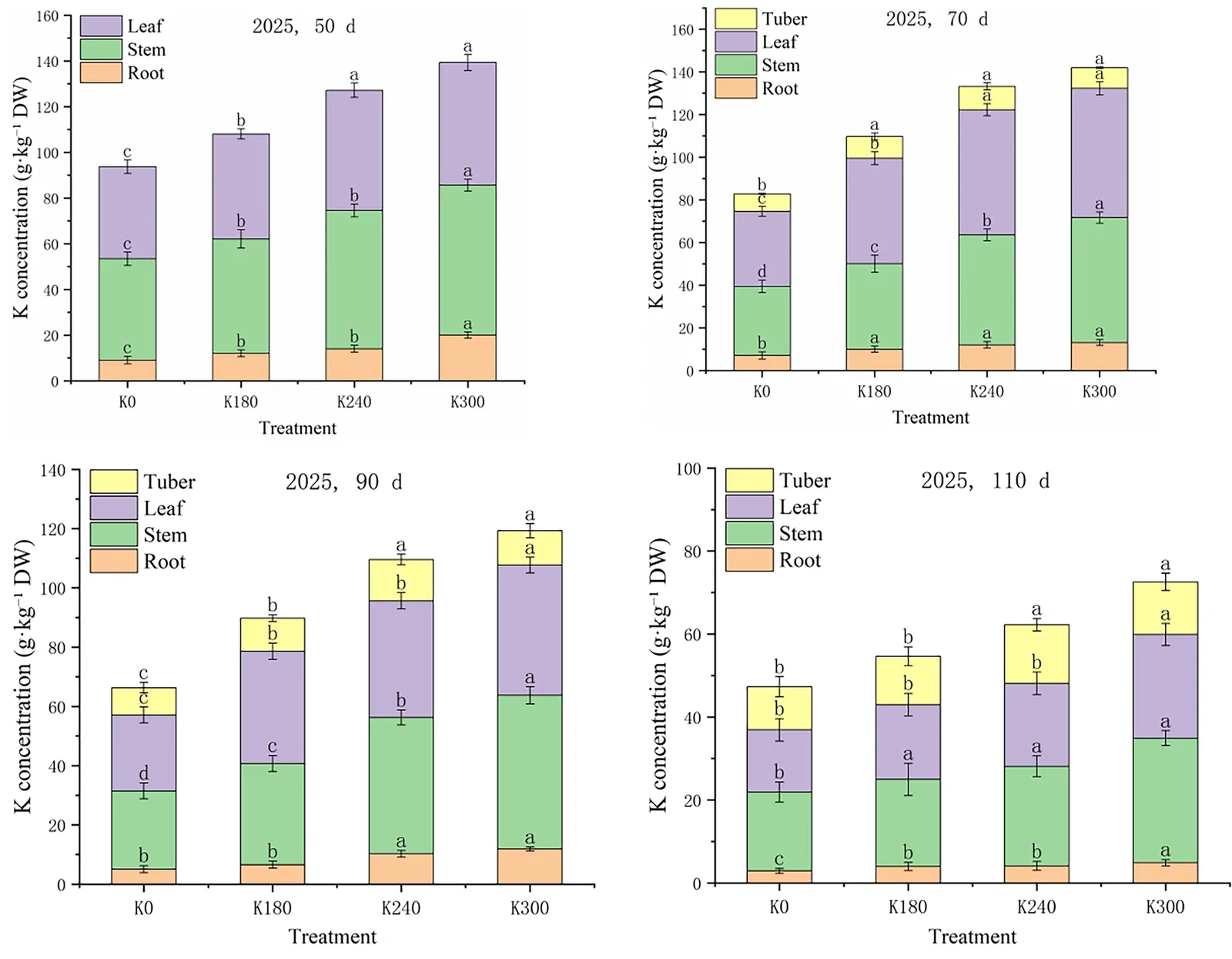
Effects of different potassium application rates on potassium concentration (g kg^-1^ DW) in different organs (roots, stems, leaves, and tubers) of potato in 2025. Data are expressed as mean ± SD (n=3). Different lowercase letters indicate significant differences among treatments at the same growth stage (P < 0.05, Tukey,s HSD test). DW, dry weight.

Regarding the distribution among organs, distinct patterns were observed across different growth stages. In the early growth stage (e.g., 50 d), before tuber formation, potassium was predominantly concentrated in the stems and leaves, with stem K concentration being significantly higher than that in leaves, while roots contained the lowest K concentration. As the growth period progressed (70 d, 90 d, and 110 d), tubers expanded and became the primary organ for K accumulation. Concurrently, the K concentration in stems and leaves gradually declined or stabilized, whereas the K concentration in tubers continuously increased, resulting in a notably higher proportion of potassium allocated to the tubers.

A longitudinal comparison of the sampling dates revealed that the total K concentration across plant parts reached relatively high levels during the early stage (50 d). In the middle-to-late stages (70 d to 110 d), the total height of the stacked bars fluctuated due to the redistribution of K from stems and leaves to the tubers. The variation trends observed in 2024 and 2025 were highly consistent, indicating good stability in the pattern of K distribution and allocation across the two experimental years. In this section, K concentration refers to the amount of potassium per unit dry weight of each organ (g kg^-1^ DW), which is distinct from K accumulation (g plant^-1^) presented in the following section.

#### 3.7.2 Potassium accumulation

The dynamic changes in total K accumulation per plant across the growth periods in both 2024 and 2025 are illustrated in the figures. In general, total K accumulation per plant significantly increased with higher potassium application rates. Throughout the entire growth period, the K0 treatment consistently maintained the lowest total K accumulation. In contrast, the high-K treatments (K240 and K300) exhibited the highest accumulation levels, with K240 slightly surpassing K300 in both experimental years.

Regarding temporal dynamics, total K accumulation in all treatments generally followed a single-peaked trend. It steadily increased from 50 days after planting (DAP), reached its maximum at 90 DAP, and then showed a slight decrease or stabilized by 110 DAP. For instance, in both 2024 and 2025, the K240 treatment peaked at approximately 7.5 to 7.8 g/plant at 90 DAP, which was substantially higher than the lower-K treatments. These patterns indicate that K accumulation primarily occurred during the middle growth stages, with the effect of K application persisting through the later stages (**Figure 12**).

**Figure 12:**
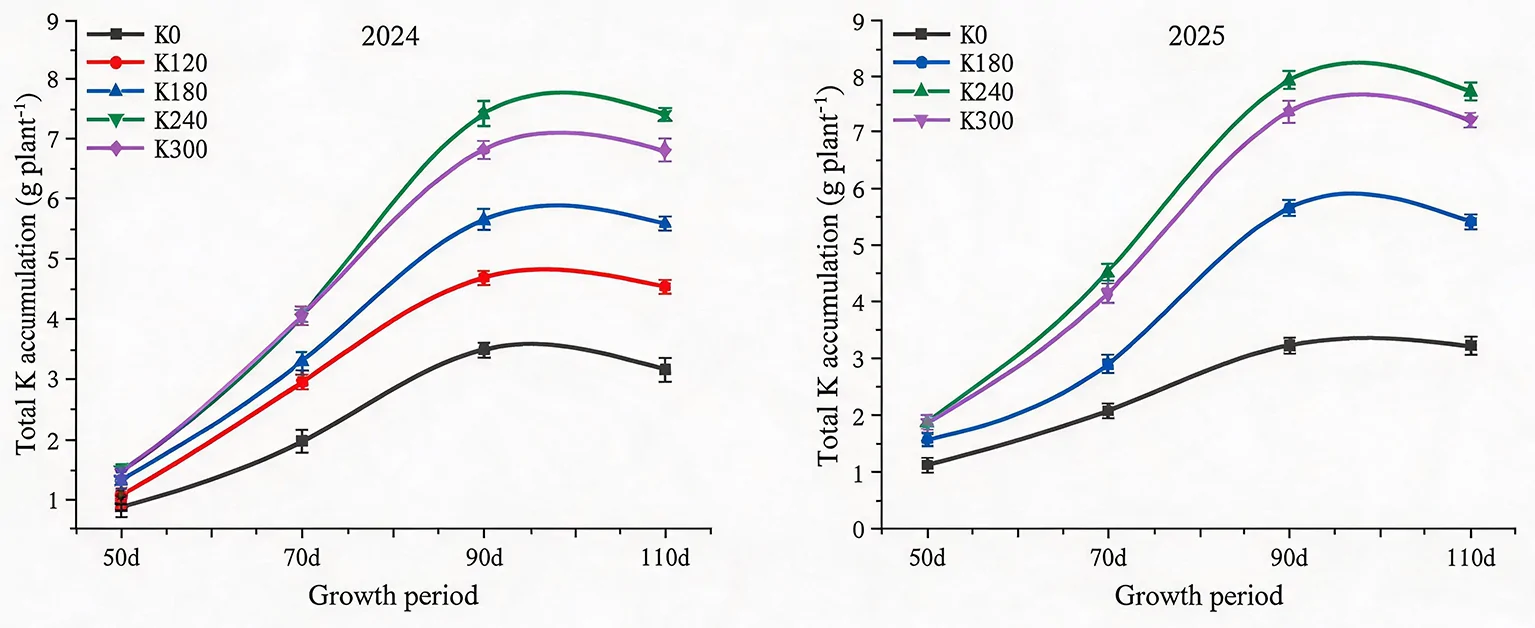
Temporal dynamics of total potassium (K) accumulation per plant under different K fertilizer treatments during the 2024 and 2025 growing seasons. The x-axis represents the growth period (50, 70, 90, and 110 days after planting), and the y-axis represents the total K accumulation (g/plant). Different lines with distinct markers denote the varying K application levels (K0, K120, K180, K240, and K300). Error bars indicate the standard deviation (SD) of the measurements.

#### 3.7.3 Potassium utilization efficiency

The potassium utilization efficiency (KUE) was significantly affected by the K application rates across both experimental years (**Figure 13**). Overall, the KUE exhibited a distinct bell-shaped response, initially increasing with rising K levels and then declining under excessive application.

**Figure 13:**
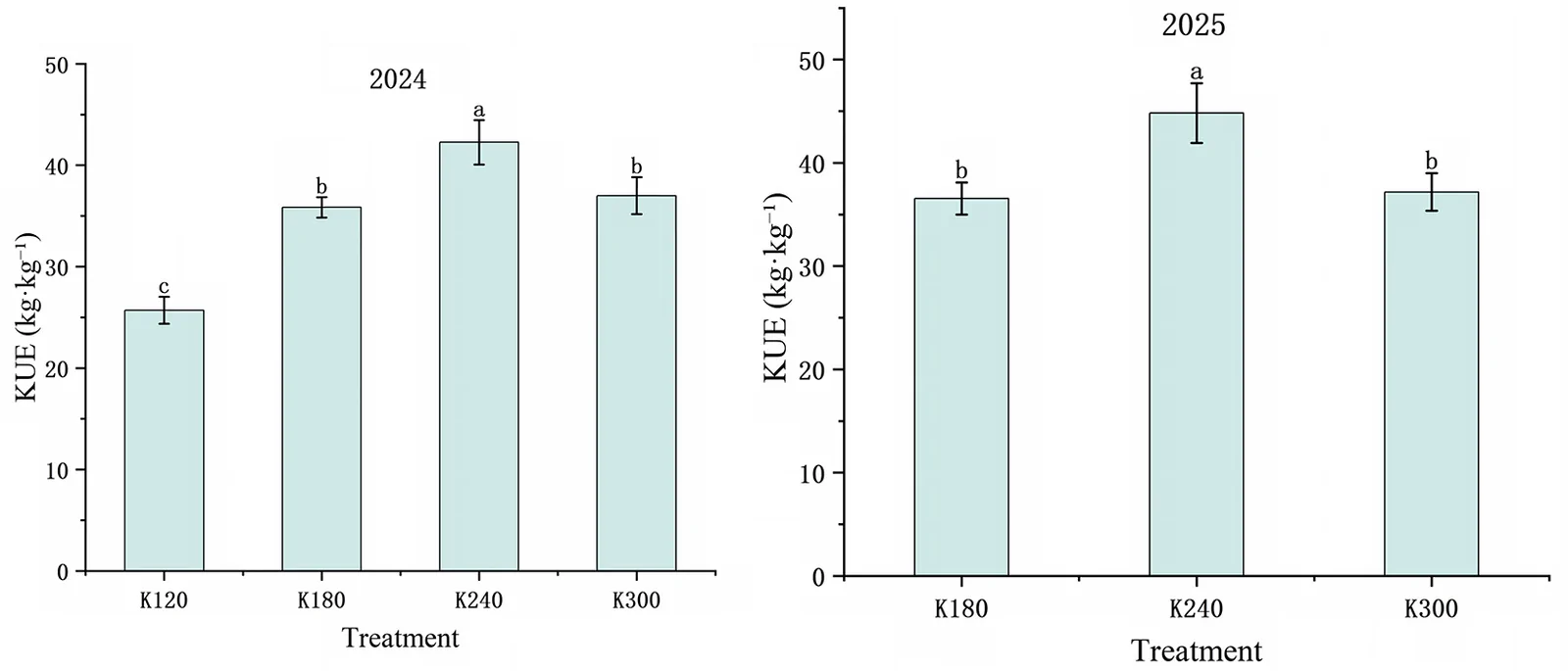
Effects of different potassium (K) fertilizer application rates on K fertilizer use efficiency (%) in the 2024 and 2025 growing seasons. The x-axis represents the K treatments (excluding the K0 control for efficiency calculation), and the y-axis indicates the K fertilizer use efficiency (%). Error bars represent the standard deviation (SD), and different lowercase letters **(a–c)** above the bars denote significant differences among the K treatments within the same year (P < 0.05).

In 2024, the KUE ranged from approximately 25 to 42 kg kg^-1^. The lowest KUE was observed under the K120 treatment, while it significantly increased at K180. The highest KUE was achieved at K240 (roughly 42 kg kg^-1^), which significantly outperformed all other treatments. However, as the application rate further increased to K300, the KUE significantly decreased compared to K240 and was statistically comparable to the K180 treatment.

In 2025, a similar trend was observed. The K240 treatment obtained the highest KUE, peaking at roughly 44 kg kg^-1^, which was significantly higher than both K180 and K300. Conversely, there was no statistically significant difference in KUE between the K180 and K300 treatments. These results indicate that moderate potassium application (K240) was optimal for maximizing potassium use efficiency.

The agronomic efficiency (AE_K) and partial factor productivity (PFP_K) of potassium fertilizer were also calculated (**Table 8**). In 2024, AE_K was highest at K180 (54.4 kg·kg^-1^), followed by K240 (49.0 kg·kg^-1^), K120 (29.9 kg·kg^-1^), and K300 (6.7 kg·kg^-1^). In 2025, AE_K was highest at K240 (43.1 kg·kg^-1^), compared with 29.3 kg·kg^-1^ at K180 and 6.5 kg·kg^-1^ at K300. Averaged across two years, AE_K followed the order: K240 (46.0 kg·kg^-1^) > K180 (41.9 kg·kg^-1^) >> K300 (6.6 kg·kg^-1^). The low AE_K at K300 confirms that excessive potassium application provides minimal additional yield return per unit of fertilizer applied.

**Table 8:** Agronomic efficiency (AE_K) and partial factor productivity (PFP_K) of potassium fertilizer under different application rates.

Year	Treatment	AE_K (kg·kg^-1^)	PFP_K (kg·kg^-1^)
2024	K120	29.9	371.7
2024	K180	54.4	282.3
2024	K240	49.0	219.9
2024	K300	6.7	143.4
2025	K180	29.3	268.1
2025	K240	43.1	222.1
2025	K300	6.5	149.7

AE_K = (yield with K − yield without K) / K_2_O application rate; PFP_K = yield / K_2_O application rate. Values are calculated from treatment means.

As expected, PFP_K decreased with increasing K application rate due to the progressive dilution of fertilizer input over the same yield base. In 2024, PFP_K declined from 371.7 kg·kg^-1^ at K120 to 143.4 kg·kg^-1^ at K300; in 2025, it declined from 268.1 kg·kg^-1^ at K180 to 149.7 kg·kg^-1^ at K300. The PFP_K values at K240 were 219.9 and 222.1 kg·kg^-1^ in 2024 and 2025, respectively, indicating that K240 maintained relatively high productivity per unit of K input while achieving the highest absolute yield.

## 4 Discussion

### 4.1 Potato yield shows a unimodal response to potassium application rate

Potato yield in our two-year field experiment exhibited a typical “low-potassium limitation and high-potassium inhibition” pattern, with the K240 treatment (240 kg·hm^-2^ K_2_O) achieving the highest yield (52, 800–53, 300 kg·hm^-2^) and a 23–29% increase over the no-potassium control. The K300 treatment did not further increase yield but showed a slight reduction trend. This result is consistent with the general understanding that potatoes have a high potassium demand but are sensitive to excess, which can cause osmotic stress and nutrient antagonism (Zhang et al., 2025).

Quadratic regression analysis indicated the non-linear nature of the yield response, with theoretical optima estimated at 181.2 and 172.9 kg·hm^-2^ for 2024 and 2025, respectively. However, as the reviewer noted, a symmetric quadratic model may not be optimal for data exhibiting a plateau response. We therefore also fitted a linear-plateau model (**Figure 4**), which estimated the breakpoint at 180.0 kg·hm^-2^ and a plateau yield of 48, 579 kg·hm^-2^, with improved goodness-of-fit (R^²^ = 0.61) compared with the quadratic model (R^²^ = 0.52). The linear-plateau analysis suggests that the breakpoint falls between K180 and K240, and that K240 lies within or near the plateau range. While K180 approached the plateau threshold in 2024, K240 consistently outperformed K180 in 2025, indicating that a higher rate within the plateau range provides a margin of safety against year-to-year environmental variability. Thus, rather than a single theoretical optimum, the yield response is better characterized as a rise to a plateau, with K240 representing a robust rate that ensures yields remain on the plateau across varying seasonal conditions.

Notably, while the yield difference between K180 and K240 was not significant in 2024, K240 significantly outperformed K180 in 2025, suggesting that under continuous cropping or longer-term conditions, a higher potassium supply may be needed to sustain high yields. This is consistent with the linear-plateau interpretation: K180 lies near the breakpoint, while K240 provides a safety margin that ensures yields remain on the plateau across varying seasonal conditions (Li et al., 2025) (Aytekin and Çalışkan, 2024; Mwesige et al., 2024). Based on the consistent yield superiority of K240 across both years and the linear-plateau analysis (**Figure 4**), 240 kg·hm^-2^ K_2_O is recommended for potato cultivation in the northern foothills of the Yinshan Mountains. The linear-plateau model indicates that the yield plateau begins at approximately 180 kg·hm^-2^, and K240 provides a buffer against seasonal variability while maintaining high yield potential.

The K120 treatment was tested only in 2024 because its yield did not differ significantly from the K0 control in that year, while the comparison between K180, K240, and K300 was considered more informative for validating the unimodal response pattern. The consistent trends observed across both years for the shared treatments (K0, K180, K240, K300) support the robustness of the main conclusions.

### 4.2 Response of *CIPK8* expression to potassium application and its potential association with potassium uptake

As a member of the CBL-interacting protein kinase family, *CIPK8* has been implicated in the regulation of potassium uptake and transport in other plant species (Yang et al., 2022). In potato, however, its functional role remains largely uncharacterized. Our study provides the first correlative evidence linking *CIPK8* expression to potassium responsiveness in potato. Recent genome-wide studies have identified multiple CIPK family members in potato, some of which are involved in nutrient stress responses (Ma et al., 2021; Huang et al., 2024; Liu et al., 2025). This study revealed at the molecular and cellular levels that CIPK8 in potato exhibits distinct localization patterns: fluorescence *in situ* hybridization showed that the *CIPK8* sequences are detected in the nucleus, while immunofluorescence localization showed that the CIPK8 protein is localized to the nucleus, cytoplasm, and membrane. This localization pattern is broadly consistent with the established signal transduction function of CIPK family members in other species, where CIPK proteins typically interact with membrane-localized CBL proteins to transmit signals to the nucleus, thereby modulating downstream gene expression (Batistic and Kudla, 2004).

Expression analysis revealed that the expression levels of the *CIPK8* gene and its protein followed a pattern of first increasing and then decreasing with increasing potassium application rates, indicating a concentration dependent response of *CIPK8* to potassium. Appropriate potassium application (K240) was associated with the highest CIPK8 expression levels, whereas low (K0) or excessive (K300) potassium application resulted in reduced expression. This pattern suggests that CIPK8 expression is concentration-dependently associated with potassium availability, and that K240 may represent the optimal level for CIPK8-associated signaling. However, direct evidence for enhanced potassium uptake (e.g., tissue K^+^ concentration measurements) and functional validation are needed to test this interpretation. This expression pattern is consistent with observations for other CIPK family members in Arabidopsis, where CIPK23-CBL complexes activate AKT1 and HAK5 to enhance K^+^ uptake, and excessive potassium concentrations have been shown to reduce their kinase activity (Chen et al., 2024). Whether a similar mechanism operates in potato remains to be investigated. Moreover, recent evidence in *Arabidopsis* indicates that the phosphorylation status of CBL-CIPK complexes is dynamically regulated by potassium availability, involving both kinases and phosphatases (Li et al., 2023; Nieves-Cordones et al., 2023). In summary, our results demonstrate a strong correlation between potassium application rate, CIPK8 expression, and potato yield, with K240 consistently showing the highest levels of both yield and CIPK8 expression. These findings identify CIPK8 as a candidate gene potentially involved in the potato response to potassium availability and provide a molecular basis for understanding the optimal potassium rate observed in this study. However, functional validation is required to establish a causal role for CIPK8 in potassium uptake and yield formation. Specifically, overexpression, RNAi, CRISPR/Cas9 knockout, or virus-induced gene silencing (VIGS) of StCIPK8 in potato, combined with K^+^ uptake and yield measurements, are needed to test causality. In this study, we use ‘K accumulation’ to refer to the total potassium amount per plant (g plant^-1^) determined at harvest, whereas ‘K uptake’ is used more broadly to describe the physiological process by which plants acquire potassium from the soil, particularly in the context of *CIPK8-associated* regulation.

### 4.3 Distinct subcellular localizations of CIPK8 transcript and protein suggest multiple functional pools

A notable observation of this study is the apparent differential subcellular localization of the *CIPK8* transcript versus the CIPK8 protein. Fluorescence *in situ* hybridization (FISH) detected CIPK8 probe signals in the nuclear region (**Figure 6**), whereas immunofluorescence detected CIPK8 protein in the nucleus, cytoplasm, and plasma membrane (**Figures 8**, **9**). Because fluorescence intensity was not quantified, the apparent differences in subcellular distribution between cell types should be considered qualitative and preliminary. However, because the FISH experiment was performed on fixed tissues without RNase/DNase controls, the nuclear signal may represent either genomic DNA or nascent transcripts, rather than exclusively mature mRNA. The nuclear enrichment of CIPK8 sequences, if suggested as RNA-specific, would represent an unusual pattern compared with typical cytoplasmic mRNA localization. However, the biological significance of this observation remains unclear and warrants further investigation with RNA-specific FISH controls. To our knowledge, this is the first observation of nuclear-enriched CIPK8 sequences in potato, although the methodological limitations noted above should be considered. Notably, a recent study showed that *AtCIPK20* in *Arabidopsis* localizes to cortical microtubules, demonstrating that non-membrane localization is not unique to *CIPK8* and may represent a broader functional feature of the CIPK family (Li et al., 2024). The protein’s presence in multiple compartments (membrane, cytoplasm, and nucleus) is consistent with the established role of CIPK-CBL modules in *Arabidopsis*, where they transduce calcium signals from membrane-associated sensors to nuclear transcriptional regulators (Chen et al., 2024). The apparent difference in subcellular localization between **Figure 8** (vascular bundle cells, predominantly nuclear) and **Figure 9** (mesophyll cells, nucleus, cytoplasm, and membrane) may reflect cell-type-specific distribution patterns. In vascular bundle cells, which are primarily responsible for long-distance transport, CIPK8 may predominantly function in nuclear signaling. In contrast, in mesophyll cells, which are more actively involved in metabolic processes and stress responses, CIPK8 may be distributed across multiple compartments to integrate signals from the membrane, cytoplasm, and nucleus. Such cell-type-specific protein distribution has been observed for other signaling proteins and may reflect the functional specialization of different tissues. Based on the observed multi-compartment distribution of CIPK8 protein in fixed tissues, we propose the hypothesis that CIPK8 may shuttle between the plasma membrane, cytoplasm, and nucleus to integrate potassium sensing and transcriptional regulation. However, because our localization experiments were performed on fixed tissue sections and fluorescence intensity was not quantified, this hypothesis should be considered speculative and preliminary. Live-cell imaging or biochemical fractionation approaches would be required to test this hypothesis.

### 4.4 Implications for sustainable potato production and potassium management

Our study provides both a theoretical basis and a practical recommendation for potassium fertilization in potatoes. The identification of K240 as the optimal rate (240 kg·hm^-2^ K_2_O) offers a concrete guideline for farmers in the Yinshan Mountain region and similar agroecological zones. Moreover, the molecular evidence that excessive potassium downregulates *CIPK8* expression and reduces yield underscores the importance of avoiding over-fertilization, which not only wastes resources but also risks environmental pollution (Ravensbergen et al., 2024; Wei et al., 2024).

The effectiveness of the recommended K rate is likely to depend on soil properties. In calcareous soils, such as the chestnut soil at our site, K^+^ can be fixed by 2:1 clay minerals and precipitated with CaCO_3_, reducing exchangeable K availability. The relatively high available K at our site (90 mg kg^-1^) may have buffered this effect, but in soils with stronger K fixation or lower available K, a higher application rate or split application may be needed. Conversely, in sandy soils with low cation exchange capacity (CEC), K240 may exceed crop demand and increase leaching risk. Therefore, the K240 recommendation should be validated across soil types before broad extrapolation. Site-specific K management should integrate soil texture, clay mineralogy, CEC, CaCO_3_ content, and initial available K status.

From an economic perspective, the marginal yield gain from increasing the K rate from 180 to 240 kg·hm^-2^ (approximately 10 kg yield per kg K_2_O) was substantial. The additional yield at K240 compared with K180 was sufficient to offset the extra fertilizer cost, supporting K240 as economically optimal. In contrast, the yield loss at K300 would result in a net economic loss, reinforcing the recommendation to avoid excessive potassium application.

It should be noted that in 2024, AE_K at K180 (54.4 kg·kg^-1^) was slightly higher than at K240 (49.0 kg·kg^-1^), reflecting the diminishing marginal returns of additional K input beyond the plateau threshold. However, in 2025, AE_K at K240 (43.1 kg·kg^-1^) exceeded that at K180 (29.3 kg·kg^-1^), and the two-year average AE_K was also higher at K240 (46.0 kg·kg^-1^) than at K180 (41.9 kg·kg^-1^). This inconsistency between years suggests that K180 may be more sensitive to seasonal variability, while K240 provides more stable agronomic efficiency across different environmental conditions. More importantly, K240 consistently achieved the highest absolute yield in both years, making it the preferred rate from a production perspective.

Beyond the economic justification, the molecular evidence also provides a basis for potential breeding applications. From a breeding perspective, the *CIPK8* gene could serve as a candidate for further investigation. If functional validation (e.g., via overexpression or gene editing in potato) confirms its role in potassium use efficiency, *CIPK8* may eventually become a target for marker-assisted selection or genetic improvement. At present, however, its utility in breeding remains speculative. Recent advances in potassium transporter gene editing and *CIPK* overexpression in potato (Huang et al., 2024) have validated the feasibility of targeting signaling nodes. Furthermore, a holistic understanding of the potassium-signaling network and the integration of multi-omics approaches (He et al., 2022) can accelerate the development of potassium-efficient potato cultivars, providing promising avenues for genetic improvement of potassium use efficiency. For instance, if future functional studies confirm that *CIPK8* positively regulates potassium uptake, overexpressing *CIPK8* or engineering its upstream regulatory elements might potentially allow plants to maintain high potassium uptake under moderate potassium supply, thereby reducing fertilizer input. However, this remains speculative until such experiments are performed. Future studies should evaluate the performance of *CIPK8*-modified potato lines under field conditions and across different soil types.

### 4.5 Limitations and future perspectives

Several limitations of this study should be acknowledged. First, our subcellular localization experiments were based on immunofluorescence of fixed tissue sections, which provide high spatial resolution but cannot capture the dynamic trafficking of the CIPK8 protein in living cells. Live-cell imaging or biochemical fractionation would be needed to confirm the proposed shuttling of CIPK8 between the cell nucleus (where its transcript localizes) and the membrane-cytoplasm-nucleus compartments (where the protein is detected). The FISH results should be interpreted with caution because no-probe, sense-probe, and RNase/DNase controls were lacking. The nuclear signal therefore cannot be definitively assigned to mature mRNA rather than genomic DNA or nascent transcripts. In addition, the FISH signal was observed in both the nucleus and the cytoplasm, and the apparent nuclear enrichment should be considered qualitative rather than quantitative. Future studies should include RNA-specific FISH with no-probe, sense-probe, and RNase controls to validate the subcellular distribution of CIPK8 transcripts. Second, the upstream signaling components that regulate CIPK8 transcription in response to external K^+^ concentration remain unknown, and their counterparts in potato have yet to be characterized. Third, the immunofluorescence results should also be interpreted with caution. No-primary-antibody and peptide-competition controls were not performed, so although three antibody dilutions gave consistent localization patterns, non-specific binding cannot be formally excluded. In addition, the localization data are based on fixed tissue sections and thus represent static snapshots rather than dynamic trafficking. Future studies should include no-primary and peptide-competition controls, ideally combined with live-cell imaging or biochemical fractionation, to validate the subcellular distribution of CIPK8 protein. Fourth, this study focused exclusively on yield parameters and the molecular responses of CIPK8 to potassium application. Although the introduction acknowledges the importance of tuber quality in potato production, quality-related parameters (e.g., starch, protein, vitamin C, or reducing sugar content) were not measured in the present experiment. Therefore, the conclusions regarding yield and CIPK8 expression should not be extrapolated to tuber quality traits without further targeted investigation. Future studies should incorporate quality assessments to establish whether the K240 treatment and CIPK8 expression are also associated with improved tuber quality. While the observed band patterns and staining distributions were reproducible, antibody specificity should be indicated in future studies using additional validation approaches.

It should be noted that the present study primarily establishes correlations between potassium application rates, CIPK8 expression, and yield. While our data identify CIPK8 as a strong candidate gene involved in potassium uptake regulation, causal evidence—such as from gene overexpression, RNA interference, CRISPR/Cas9 knockout, or virus-induced gene silencing (VIGS)—is required to definitively establish its regulatory role in potassium uptake and yield formation. Therefore, the conclusions regarding CIPK8-mediated regulation should be interpreted as hypotheses generated by this study, warranting further functional validation.

The subcellular localization data presented in this study are based on immunofluorescence of fixed tissue sections and thus represent static snapshots rather than dynamic trafficking information. Conclusions regarding protein shuttling or signal transduction dynamics should be considered speculative until indicated by live-cell imaging. In addition, soil K fixation capacity and clay mineralogy were not measured in this study; these properties should be included in future site-specific K recommendations. Because the K240 recommendation is based on a single site and two years, its extrapolation to soils with different K fixation capacities, textures, or initial available K levels should be made with caution.

Because fluorescence intensity was not quantified, the apparent differences in subcellular distribution between cell types (vascular bundle cells vs. mesophyll cells) should be considered qualitative and preliminary. The FISH and immunofluorescence data therefore provide suggestive, rather than definitive, evidence for the subcellular distribution of CIPK8 transcript and CIPK8 protein.

Future research should address: (i) the transcriptional regulators of *CIPK8* (e.g., transcription factors binding to its promoter); (ii) the exact post-translational modifications and interacting partners of *CIPK8* in potato; and (iii) whether the cell-nucleus localization of *CIPK8* mRNA has a functional role beyond translation. Addressing these questions will further refine potassium management strategies and deepen our understanding of K^+^ sensing in crops.

## 5 Conclusion

In this study, using the potato cultivar ‘V7’ as the material, a two-year field experiment was conducted with different potassium application rates (0, 120, 180, 240, and 300 kg·hm^-2^ K_2_O) to investigate the effects of potassium application rate on yield and to characterize the expression patterns of CIPK8, a candidate gene potentially involved in potassium uptake regulation. The main conclusions are as follows:

### 5.1 Appropriate potassium application significantly increases potato yield

Among the tested rates, K240 (240 kg·hm^-2^ K_2_O) consistently produced the highest yield in both years (52, 800–53, 300 kg·hm^-2^), with increases of 23–29% over the no-potassium control. Quadratic response analysis confirmed the unimodal yield pattern, with excessive potassium (>240 kg·hm^-2^) leading to yield reduction.

### 5.2 K240 was associated with the highest CIPK8 gene and protein expression, peaking at the tuber bulking stage (90 days)

These results identify CIPK8 as a candidate responsive gene, not a confirmed regulator.

### 5.3 FISH detected *CIPK8* sequences in the nucleus, while immunofluorescence detected CIPK8 protein in the nucleus, cytoplasm, and cell membrane

Because fluorescence intensity was not quantified, this differential distribution pattern should be considered qualitative and preliminary, and the FISH results require confirmation with RNA-specific controls.

### 5.4 Based on the two-year field results, K240 (240 kg·hm^-^² K_2_O) was consistently the highest-yielding treatment among those tested and is recommended for potato production in the northern foothills of the Yinshan Mountains and similar agroecological zones

The linear-plateau analysis indicated that the yield response reaches a plateau within the 180–240 kg·hm^-^² range, and K240 is preferred as it provides a buffer against seasonal variability while maintaining high yield potential. This rate was associated with the highest yield and the highest CIPK8 expression, identifying CIPK8 as a candidate gene responsive to potassium application. Functional validation of CIPK8 is required before it can be considered a regulatory target. This study provides a molecular basis for efficient potassium utilization in potato. The K240 recommendation should be validated across soil types, particularly those differing in K fixation capacity and clay mineralogy.

## Data Availability

The original contributions presented in the study are included in the article/**Supplementary Material**. Further inquiries can be directed to the corresponding author.
